# Mutasynthesis and Antibiotic Activity of Mupirocin Analogues

**DOI:** 10.1002/cbic.70439

**Published:** 2026-06-30

**Authors:** Sarah M. Husain, Luoyi Wang, Li‐Chen Han, James I. Bowen, Zhongshu Song, Felix de Courcy‐Ireland, Ashley J. Winter, Thomas J. Simpson, Paul R. Race, James Spencer, Matthew P. Crump, Christine L. Willis

**Affiliations:** ^1^ School of Chemistry, Cantock's Close University of Bristol Bristol UK; ^2^ Department of Chemistry, Faculty of Science Taibah University Madinah Saudi Arabia; ^3^ Institute of Microbiology Chinese Academy of Sciences Beijing China; ^4^ School of Natural and Environmental Sciences Newcastle University Newcastle upon Tyne UK; ^5^ School of Cellular and Molecular Medicine University of Bristol Bristol UK

**Keywords:** antimicrobial, *Bacillus subtilis*, biosynthesis, metabolic engineering, mupirocin, polyketide, *Pseudomonas fluorescens*, synthesis

## Abstract

With growing understanding of complex biosynthetic pathways to natural products, mutasynthesis, which combines metabolic engineering with chemical synthesis, is becoming an increasingly important tool to produce novel compounds. Mupirocin, isolated from *Pseudomonas fluorescens*, is a mixture of pseudomonic acids (PAs) that exhibit antibiotic activity against Gram‐positive bacteria including methicillin‐resistant *Staphylococcus aureus*. We have developed a flexible approach, based on mutasynthesis, for the preparation of a library of novel PA analogues. The antimicrobial activities of the natural products and synthetic analogues were evaluated against *Bacillus subtilis* and four *Staphylococcus aureus* strains. Interestingly, one of the analogues retained antimicrobial activity in all the assays but lacked structural features that render PA‐A unstable, that is, the 10,11‐epoxide (replaced by an alkene) and ester linkage (replaced by a ketone). In addition, mutasynthesis allowed the preparation of an analogue of a key biosynthetic intermediate (desepoxy‐PA‐B) in which the C_9_ hydroxy fatty acid is replaced by a C_7_ analogue. Feeding studies with mutant strains of *Pseudomonas fluorescens* revealed that C_7_‐desepoxy‐PA‐B was converted to the novel metabolite C_7_‐PA‐C with loss of the 8‐hydroxyl group but with no extension to the C_9_ side chain.

## Graphical abstract

A mutasynthetic strategy was developed to prepare pseudomonic acid analogues, and their antimicrobial activities were evaluated against five bacterial strains. Feeding studies with mutant strains of *Pseudomonas fluorescens* revealed that C_7_‐desepoxy‐PA‐B was metabolised to C_7_‐PA‐C with loss of the 8‐hydroxyl group, but no fatty acid chain extension occurred.

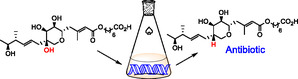

## 1 Introduction

**SCHEME 1:**
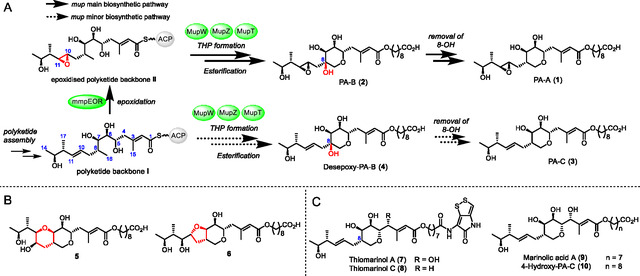
(A) Outline of the later stages of mupirocin biosynthesis in *Pseudomonas fluorescens*. The major pathway proceeds via epoxidation of the 10,11‐alkene, giving PA‐A (**1**) as the major metabolite. A minor parallel pathway generates metabolites with a 10,11‐alkene leading to the formation of PA‐C (**3**) (AC*P* = acyl carrier protein). (B) Rearrangement products **5** and **6** from the treatment of PA‐A (**1**) with acid. (C) Thiomarinols and metabolites lacking the pyrrothine from *Pseudoalteromonas* strains.

Mupirocin was first isolated from *Pseudomonas fluorescens* NCIMB 10 586 and is used clinically as an antibiotic against Gram‐positive bacteria, including methicillin‐resistant *Staphylococcus aureus* (MRSA) [1, 2]. It inhibits the bacterial isoleucyl tRNA synthetase enzyme responsible for loading the amino acid isoleucine (Ile) onto its cognate tRNA required for ribosomal protein synthesis [3]. Mupirocin comprises a mixture of metabolites, with the major component (ca 90%) being pseudomonic acid A (PA‐A, **1**), consisting of a C_17_‐polyketide‐derived moiety esterified by 9‐hydroxynonanoic acid (9HN) (Scheme 1A). Further minor components of mupirocin include PA‐B (**2**) with an additional hydroxyl group at C‐8, and PA‐C (**3**) with a 10,11‐alkene instead of the epoxide. The 10,11‐epoxide renders PA‐A (**1**) unstable outside a narrow pH range due to attack of the 7‐hydroxyl group on the oxirane ring, generating bicyclic oxygen heterocycles **5** and **6,** which are biologically inactive (Scheme 1B) [4, 5]. This limits the clinical use of mupirocin to topical application or nasal administration as an anti‐MRSA agent [6, 7].

Mupirocin is biosynthesised by a hybrid *trans*‐acyltransferase (*trans*‐AT) polyketide synthase; gaining an understanding of its complex biosynthetic pathway has presented a significant challenge [8, 9]. A combination of biochemical and chemical investigations, including mutagenesis experiments, has revealed that assembly of the heptaketide backbone **I** occurs on two large multi‐modular proteins with MupA‐catalysed addition of the 6‐hydroxyl group [10] as well as formation of the β‐branch at C‐3 by the hydroxymethylglutaryl‐CoA synthase (HMGS) cassette (Scheme 1A) [11]. From the linear polyketide intermediate **I,** the precise sequence of biosynthetic steps leading to PA‐B (**2**) is not fully elucidated, but includes: 10,11‐epoxidation by MmpEOR to **II** [12, 13]; formation of the tetrahydropyran (THP) ring by the combined action of the oxygenase MupW, its redox partner MupT, and an epoxide hydrolase MupZ [14, 15]; and esterification leading to the 9‐hydroxynonanoate [2, 16]. PA‐B (**2**) is converted to the major product PA‐A (**1**) through a series of transformations, which ultimately lead to the removal of the 8‐hydroxyl group [17, 18]. Desepoxy‐PA‐B (**4**) and PA‐C (**3**) are minor metabolites formed by a parallel series of reactions from intermediate **I** and are more stable than their counterparts that contain a 10,11‐epoxide. Cultures of the *mmpE*Δ*OR* mutant strain of *P. fluorescens*, which lacks the 10,11‐epoxidase, produce PA‐C (**3**) as the major metabolite [12].

The closely related thiomarinols (e.g. **7** and **8**) are a family of marine natural products isolated from *Pseudoalteromonas* sp. SANK 73 390, which also exhibits potent activity against MRSA [19]. They all possess a 10,11‐alkene and are assembled on a similar C_17_‐polyketide moiety to PA‐C (**3**) but esterified with an 8‐hydroxyoctanoic acid (Scheme 1C) [20]. The octanoic acid is further linked via an amide bond to the NRPS/cysteine‐derived pyrrothine heterocycle, and the Δ*NRPS* mutant of *Pseudoalteromonas* accumulates marinolic acids (e.g. **9**) lacking this modification [21, 23]. TmuB has been identified as the additional α‐ketoglutarate‐dependent dioxygenase responsible for the 4‐hydroxylation in thiomarinol biosynthesis [24]. Introduction of the *tmuB* gene from the *Pseudoalteromonas* sp. SANK 73 390 strain into the *mmpE*Δ*OR* mutant of *P. fluorescens* NCIMB 10 586 gave the *mmpE*Δ*OR‐tmuB* variant, which produced 4‐hydroxy PA‐C (**10**) as the major metabolite [24].

Herein, we report the use of mutasynthesis to prepare a panel of PA‐C analogues and investigate their antimicrobial activities, along with those of the known 4‐hydroxylated counterparts **7**, **9,** and **10**, against *Bacillus subtilis* and four *Staphylococcus aureus* strains, including the methicillin‐resistant and glycopeptide‐intermediate Mu50 strain. In addition, mutasynthesis proved valuable in the preparation of an analogue of a key biosynthetic intermediate, desepoxy‐PA‐B (**4**), which was used in feeding studies with mutant strains of *P. fluorescens* to give further insight into mupirocin biosynthesis and provide a novel metabolite for biological assessment.

## 2 Results and Discussion

**SCHEME 2:**
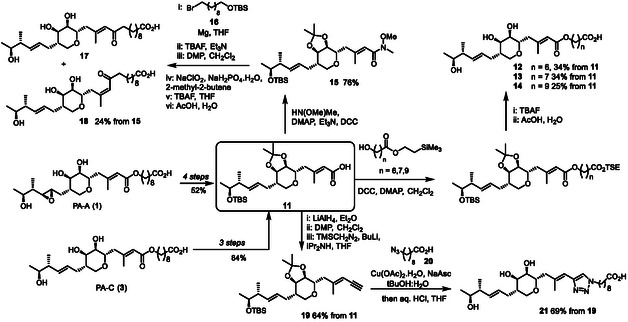
Summary of synthetic routes to PA‐C analogues linked via an ester to different length fatty acid side chains **12**, **13** and **14,** ketones **17** and **18,** and triazole **21**. DCC = *N*,*N’*‐dicyclohexylcarbodiimide, DMA*P* = 4‐dimethylaminopyridine, DM*P* = Dess‐Martin periodinane, NaAsc = sodium ascorbate, TBAF = tetrabutylammonium fluoride, TSE = trimethylsilylethyl, TBS = *tert*butyldimethylsilyl.

Mupirocin acts as an antibiotic by inhibiting the bacterial isoleucyl tRNA synthetase (IleRS) [3]. The crystal structure of IleRS complexed with tRNA^ile^ and PA‐A (**1**) indicated that the 9HN side chain sits in a hydrophobic groove alongside the loop bearing the signature KMSKS motif, which may stabilise the complex [25, 26]. To examine the effect of fatty acid chain length on bioactivity, we focused on synthetic analogues of the more stable PA‐C (**3**), which contains the 10,11‐alkene and has similar activity to PA‐A (**1**), which features the labile 10,11‐epoxide. Initially, the key synthetic intermediate protected monic acid **11** was prepared in four steps from the major metabolite PA‐A (**1**) isolated from cultures of wild‐type *P. fluorescens*, by modification of an approach by Clayton et al. (Scheme 2) [5]. Protection of the *syn* diol of PA‐A (**1**) as an acetonide, deoxygenation of the epoxide with KHCO_3_ and KSeCN, silyl protection of the 13‐OH, and ester hydrolysis gave **11** in four steps and 52% yield from the natural product. However, a more efficient and sustainable strategy was to use mutasynthesis and begin with PA‐C (**3**), thus avoiding the deoxygenation step. While **3** is a minor metabolite in wild‐type *P. fluorescens*, engineering the pathway to give a hybrid mutant *mmpE*Δ*OR/*Δ*mupW‐tmlW*, in which the Rieske oxygenase (TmlW) from the thiomarinol pathway replaced MupW from the mupirocin pathway, reliably delivered 150 mg/L of PA‐C (**3**) after optimisation of the culture conditions (see Supporting Information) [27]. Coupling the protected C_17_‐polyketide moiety **11** with three trimethylsilylethyl (TSE) hydroxy‐esters with varying chain lengths, followed by a two‐step deprotection using TBAF then AcOH, gave analogues **12**, **13** and **14** with C_7_, C_8_ and C_10_ side chains, respectively.

Ester hydrolysis of pseudomonic acid (PA) to monic acid occurs under mildly basic conditions (e.g. 0.3 M aqueous NaOH) and was of value in the synthesis of acid **11** (Scheme 2). Hydrolysis also occurs in body fluids, rendering mupirocin inactive and restricting it to topical application [7, 28]. A possible way to improve the stability of PA analogues would be to substitute the ester for a ketone. A series of aryl and heteroaryl C‐1 ketone derivatives of monic acid have been prepared previously, and several displayed antibiotic activities [29, 30]. However, C‐1 ketone analogues of the PAs with a fatty acid side chain have not been reported. Hence, the novel ketones **17** and **18** were our next synthetic targets. First, conversion of acid **11** to Weinreb amide **15**, followed by addition of the Grignard reagent derived from bromide **16**, gave the required skeleton of the synthetic targets. Following selective deprotection of the silyl ether to the corresponding primary alcohol, a two‐stage oxidation using Dess‐Martin periodinane (DMP) followed by a Pinnick oxidation gave the carboxylic acid, which, after deprotection, provided a 2:1 mixture of *E* and *Z* enones **17** and **18**. Monic acid analogues in which the ester was replaced by a nitrogen heterocycle are known, but none contained a fatty acid side chain [31]. Hence, the final compound selected for biological assessment was the non‐hydrolysable triazole **21**. This was prepared from acid **11** via a two‐step procedure to generate the corresponding aldehyde, which was treated with TMS‐diazomethane in the presence of nBuLi to give alkyne **19**. A click reaction between **19** and azide **20** gave triazole **21** in 69% yield over the 2 steps.

The antimicrobial activities of the natural products and synthetic analogues were evaluated against *Bacillus subtilis* and four strains of *Staphylococcus aureus* in disc diffusion assays (Table 1). Disc diffusion is an established method for testing *S. aureus* mupirocin susceptibility, and compared to broth dilution, minimal inhibitory concentration assays consume far smaller amounts of compound. This allowed testing of our panel of analogues against multiple bacterial strains. *S. aureus* strains tested were ATCC 29 213 (a widely used standard in clinical antibacterial susceptibility testing) [32]; Newman (originally isolated from a human infection but since used extensively in animal disease models) [33]; NCTC 6571 (for penicillin sensitivity testing) [34] and Mu50 (a methicillin‐resistant strain isolated from a human patient that also shows reduced susceptibility to the glycopeptide antibiotic vancomycin) [35]. PA‐A (**1**) and PA‐C (**3**) display similar activities in all assays, while metabolites with an additional 8‐hydroxyl group, PA‐B (**2**) and desepoxy‐PA‐B (**4**), are less active (Table 1). PA‐C analogues with the C_8_ and C_10_ side chains (**13** and **14**) showed similar activity, and both were slightly more active than the C_7_‐analogue **12.** However, PA‐C (**3**) with the natural C_9_ chain remained the most potent among these four compounds. Comparing the activity of PA‐C (**3**) with 4‐hydroxy‐PA‐C (**10**), and C_8_‐PA‐C (**13**) with marinolic acid A (**9**) revealed that 4‐hydroxylation reduced activity. In contrast, in the case of the thiomarinols, 4‐hydroxylation increases potency, as seen when comparing the activities of thiomarinol A (**7**) and thiomarinol C (**8**). This result is consistent with previous observations that 4‐hydroxylation has opposite effects on the respective potencies of mupirocin and thiomarinol [24].

**TABLE 1:** Antibiotic activities of natural products and analogues from disk diffusion assays.

*Compound*	*Bacillus subtilis*	*Staphylococcus aureus*			
		*ATCC 29 213*	*Newman*	*NCTC 6571*	*Mu50*
PA‐A (**1**)	15.7 ± 1.2	16.7 ± 0.6	16.3 ± 1.5	17.0 ± 0	20.0 ± 1.0
PA‐B (**2**)	/	8.7 ± 0.6	9.3 ± 1.2	11.0 ± 0.6	11.0 ± 1.0
PA‐C (**3**)	16.7 ± 0.6	16.3 ± 0.6	14.0 ± 0	16.0 ± 0	18.3 ± 1.2
DesPAB (**4**)	/	/	/	8.3 ± 0.6	/
Thio A (**7**)	23.0 ± 1.0	26.0 ± 0	28.3 ± 0.6	25.0 ± 0.6	31.0 ± 1.0
Thio C (**8**)	19.7 ± 0.6	22.7 ± 0.6	22.7 ± 0.6	21.0 ± 0	24.3 ± 1.2
Mar A (**9**)	7.7 ± 0.6	10.7 ± 0.6	8.7 ± 0.6	9.7 ± 0.6	11.3 ± 1.2
4OH PAC (**10**)	12.0 ± 1.0	12.3 ± 0.6	11.7 ± 0.6	12.0 ± 0.6	16.7 ± 1.2
C_7_‐PAC (**12**)	9.0 ± 1.0	12.0 ± 1.7	10.3 ± 0.6	14.0 ± 0	13.3 ± 0.6
C_8_‐PAC (**1** **3**)	12.3 ± 0.6	13.3 ± 1.2	10.7 ± 0.6	13.0 ± 1.0	15.0 ± 1.0
C_10_‐PAC (**1** **4**)	13.3 ± 2.9	13.7 ± 0.6	11.7 ± 0.6	13.0 ± 0	15.0 ± 0
*E*‐ketone (**17**)	13.7 ± 0.6	12.3 ± 0.6	13.7 ± 0.6	14.0 ± 0.6	15.3 ± 1.2
*Z*‐ketone (**18**)	11.7 ± 0.6	11.0 ± 1.0	10.0 ± 0	13.0 ± 0.6	13.0 ± 1.7
Triazole (**21**)	/	/	/	/	/

*Note:* 3 μL of a 100 μg/mL methanol solution of the compounds was added to the discs. Each compound was tested in triplicate, and activities are quoted as mean ± standard deviation. The discs measured 5 mm in diameter. All controls (methanol) were negative. / Indicates no observable activity. Plates were incubated overnight at 37°C in ambient air, and zones of growth inhibition were read after 18‐h incubation.

Finally, attention was turned to analogues **17**, **18** and **21** that lack the ester which unites the fatty acid side chain with the polyketide fragment of PAs and thiomarinols. Triazole **21** was inactive in all assays. Ketones **17** and **18** were difficult to separate and so were tested as mixtures of isomers, **17** as a 2:1 mixture of *E*: *Z*−2,3‐alkenes and **18** as a 2:1 mixture of *Z*:*E*‐alkenes. Pleasingly, in both cases, they retained activity approaching that of PA‐C (**3**). These findings have potential value in future development of a more stable analogue of mupirocin, as two structural features of PA‐A (**1**), which render it unstable, have been modified:, that is the 10,11‐epoxide has been replaced by an alkene, and the unsaturated ester linking the polyketide moiety to the fatty acid side chain is now an unsaturated ketone.

**SCHEME 3:**
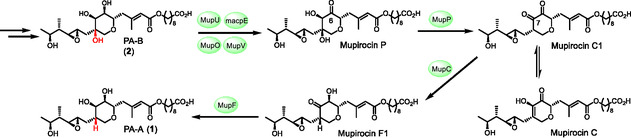
Late‐stage biosynthetic steps showing the conversion of PA‐B (**2**) to PA‐A (**1**) in *P. fluorescens*.

All mupirocins/PAs isolated from wild‐type *P. fluorescens* possessing a THP ring are also esterified with the full‐length 9‐hydroxynonanoate side chain (Scheme 3). However, the precise timing and mechanism of esterification are not proven [12]. The first biosynthetic intermediate assembled on a THP ring is PA‐B (**2**). Subsequent oxidation of **2** forms the 6‐ketone mupirocin P; dehydration effects the removal of the 8‐hydroxyl group, giving mupirocin C. Two reductive steps generate PA‐A (**1**) via mupirocin F1.

**SCHEME 4:**
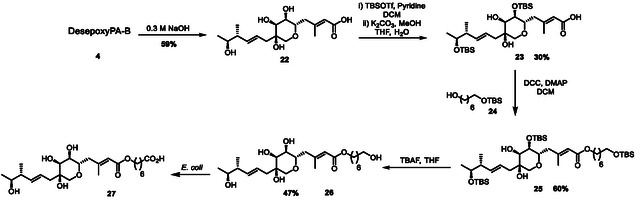
Synthesis of C_7_‐desepoxy‐PA‐B **27**.

An alternative strategy to access mupirocin analogues is via feeding substrates to mutant strains of *P. fluorescens*. C_7_‐desepoxy‐PA‐B (**27**) was selected as a substrate to demonstrate the potential value of such an approach, as it was expected to reveal if fatty acid chain extension, as well as further metabolic processing steps to remove the 8‐hydroxyl group, would occur in cultures of *P. fluorescens* (Scheme 4). To begin, desepoxy‐PA‐B (**4**) was isolated from cultures of the *mmp*Δ*EOR/*Δ*mupU‐pJH2* mutant of *P. fluorescens* in titres of 200 mg/L [27]. Following ester hydrolysis, acid **22** was treated with excess TBSOTf, and the resultant TBS ester hydrolysed, giving diTBS ether **23** as the major product. The ^1^H‐NMR spectrum of **23** showed characteristic signals for 5‐H, 6‐H and 7‐H, in accord with the THP in a chair conformation and the equatorial 6‐hydroxyl group having been converted to a silyl ether. Coupling acid **23** with alcohol **24** in the presence of DCC and DMAP, followed by global deprotection of **25** with TBAF, gave **26**. To complete the synthesis of **27**, a selective oxidation of the primary alcohol was achieved via bio‐oxidation in *Escherichia coli* BL21(DE3) cells for 2 days, giving the target acid **27** as the sole product.

**SCHEME 5:**
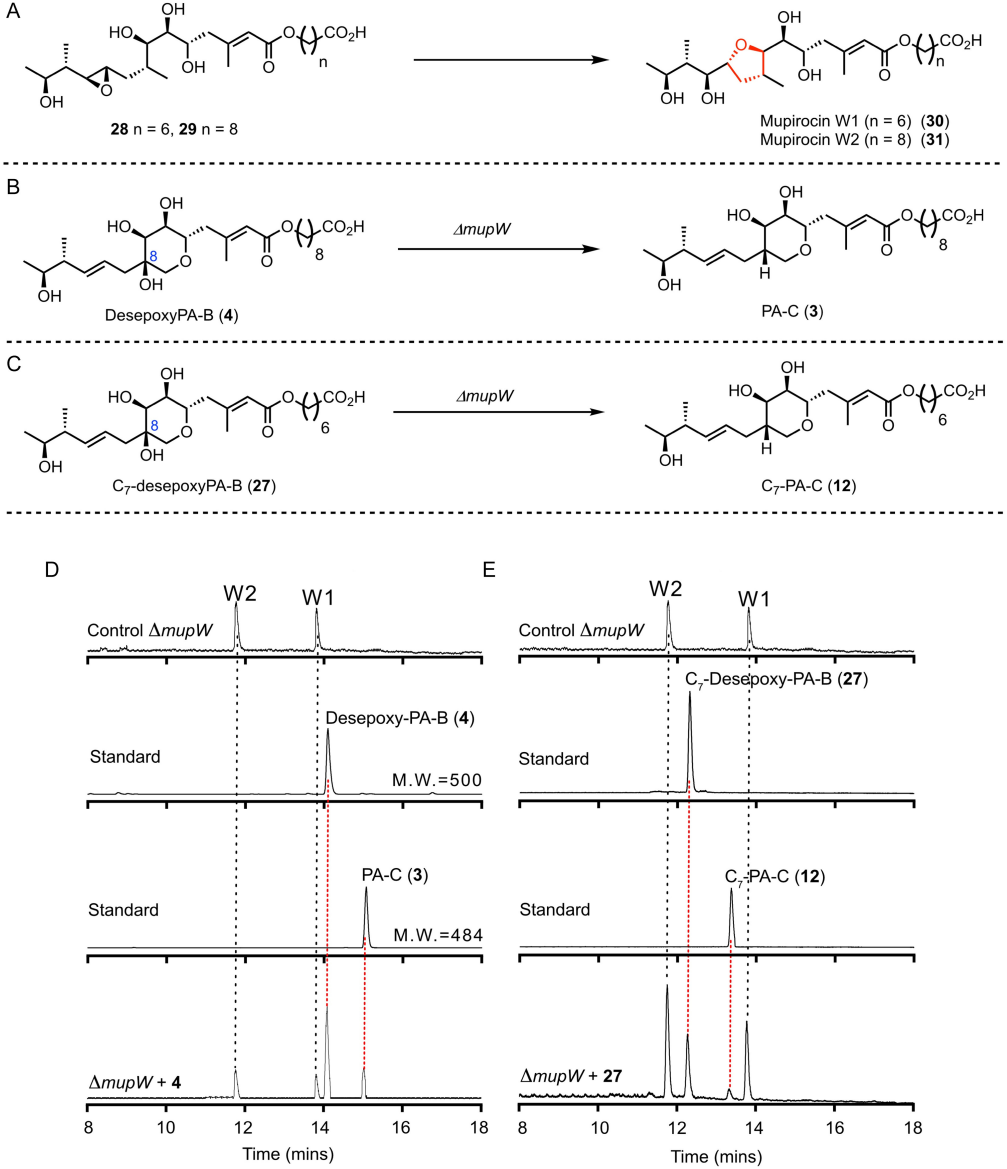
Feeding studies to the Δ*mupW* strain of *P. fluorescens*. (A) Mupirocins W1 and W2 (**30**) and (**31**) isolated from cultures of Δ*mupW* (D and E, top LC‐MS traces). (B) Feeding desepoxy PA‐B (**4**) gave PA‐C (**3**)**.** (C) Feeding C_7_‐desepoxyPA‐B (**27**) gave C_7_‐PA‐C (**12**). (D) LC‐MS trace showing conversion of **4** to **3**. (E) LCMS trace showing conversion of **27** to **12**.

Previous experiments have revealed that cultures of the Δ*mupW* mutant of *P. fluorescens* produce no metabolites with a THP ring, as the step involving oxidation of the 8‐methyl group by MupW is blocked [14]. Interestingly, two products were isolated from these cultures, one esterified with a C_7_ and the other with a C_9_ fatty acid **30** and **31,** respectively (Scheme 5A) [12]. In each case, the tetrahydrofuran ring is believed to be formed by attack of the 7‐hydroxyl group on the 10,11‐epoxide of the precursors **28** and **29**. In a control experiment, desepoxy‐PA‐B (**4**) was fed to the Δ*mupW* strain of *P. fluorescens* and was transformed to PA‐C (**3**) with overall loss of the 8‐hydroxyl group as observed by LC‐MS and confirmed by ^1^H‐NMR spectroscopy of the purified product, in accord with previous observations [12] (Scheme 5B,D). Next, C_7_‐desepoxy‐PA‐B (**27**) was fed to the mutant under the same conditions and transformed to C_7_‐PA‐C (**12**), albeit less efficiently than the natural substrate (Scheme 5C,E). No elongation of the C_7_‐heptanoate to the C_9_‐nonanoate was detected.

## 3 Conclusions

In conclusion, using a combination of mutasynthesis and organic synthesis, a flexible approach for the preparation of a small library of novel PA analogues has been developed. Evaluating antimicrobial activities of PAs and analogues against *Bacillus subtilis* and four *Staphylococcus aureus* strains, including patient‐derived and antibiotic‐resistant strains, revealed that those esterified with a 9‐hydroxynonanoic acid were more active than those with either a shorter (**12** and **13**), or longer (**14**) side chain; the presence of either a 4‐ or 8‐hydroxyl group reduced bioactivity (compounds **9**, **10** and **4**); while substitution of the C‐1 ester for a triazole motif (**21**) eradicated bioactivity. Interestingly, ketones **17** and **18** retained antimicrobial activity in all the assays but lacked structural features of PA‐A (**1**), which render it unstable, that is the 10,11‐epoxide (replaced by an alkene) and ester linkage (replaced by a ketone). These structural modifications may pave the way for wider applications of mupirocin antibiotics. Mutasynthesis was used in the preparation of the novel analogue C_7_‐desepoxy‐PA‐B (**27**) esterified with a C_7_ rather than the C_9_ hydroxy fatty acid of the natural product. Feeding studies with mutant strains of *Pseudomonas fluorescens* revealed that while C_7_‐desepoxy‐PA‐B (**27**) was converted to the novel metabolite C_7_‐PA‐C (**12**) with loss of the 8‐hydroxyl group, no chain extension to the 9‐hydroxynonanoate was apparent. Elucidating the precise mechanistic steps of esterification is the focus of ongoing investigations.

## Funding

This work was supported by Biotechnology and Biological Sciences Research Council (BB/L01386X/1, BB/W008823/1, BB/R007853/1, and BB/T001968/1) and Engineering and Physical Sciences Research Council (EP/S024107/1).

## Conflicts of Interest

The authors declare no conflicts of interest.

## Supporting information

Susceptibility testing followed Clinical Laboratory Standards Institute (CLSI) guidelines. Briefly, bacterial strains were grown overnight on Mueller‐Hinton agar (Becton‐Dickinson); 3‐5 colonies were resuspended in phosphate‐buffered saline (PBS) to a turbidity equivalent to a 0.5 MacFarland standard, and a fresh Mueller‐Hinton agar plate was inoculated with the suspension using a sterile cotton swab. 3 μL of a 100 μg/mL solution of the tested compound, dissolved in 100% methanol, was added to a sterile 5 mm filter paper disc, which was allowed to dry before application to the plate. Plates were incubated overnight at 37°C in ambient air and zones of growth inhibition read after 18 h incubation. Further supporting information can be found online in the Supporting Information Section. Supporting Information includes details of the isolation of natural products from mutant strains of *P. fluorescens*, synthetic procedures and characterisation of products by spectroscopic methods and copies of ^1^H‐ and ^13^C‐NMR spectra. The authors have cited additional references within the Supporting Information.

## Data Availability

The data that supports the findings of this study are available in the Supporting Information of this article.
