# Supplementary material for: Mutasynthesis and Antibiotic Activity of Mupirocin Analogues
Source: Chembiochem. 2026 Jun 30;27(13):e70439. doi: 10.1002/cbic.70439 (PMC13316724; doi:10.1002/cbic.70439)
Supplement: Supplementary file 1 — Susceptibility testing followed Clinical Laboratory Standards Institute (CLSI) guidelines. Briefly, bacterial strains were grown overnight on Mueller‐Hinton agar (Becton‐Dickinson); 3‐5 colonies were resuspended in phosphate‐buffered saline (PBS) to a turbidity equivalent to a 0.5 MacFarland standard, and a fresh Mueller‐Hinton agar plate was inoculated with the suspension using a sterile cotton swab. 3 μL of a 100 μg/mL solution of the tested compound, dissolved in 100% methanol, was added to a sterile 5 mm filter paper disc, which was allowed to dry before application to the plate. Plates were incubated overnight at 37°C in ambient air and zones of growth inhibition read after 18 h incubation. Further supporting information can be found online in the Supporting Information Section. Supporting Information includes details of the isolation of natural products from mutant strains of P. fluorescens, synthetic procedures and characterisation of products by spectroscopic methods and copies of 1H‐ and 13C‐NMR spectra. The authors have cited additional references within the Supporting Information. [file CBIC-27-e70439-s001.pdf]

Supplementary Information

Mutasynthesis and Antibiotic Activity of Mupirocin Analogues

Sarah M. Husain,[1,2,#] Luoyi Wang,[1,#] Li-Chen Han,[1,#] James I. Bowen,[1] Zhongshu Song,[1] Felix de Courcy-Ireland,[1] Ashley J. Winter,[1] Thomas J. Simpson,[1] Paul R. Race,[3] James Spencer,[4] Matthew P. Crump [1*] and Christine L. Willis [1*]

[1] School of Chemistry, Cantock's Close, University of Bristol, BS8 1TS, UK

[2] Department of Chemistry, Faculty of Science, Taibah University, Madinah 42353, Saudi Arabia

[3] Institute of Microbiology, Chinese Academy of Sciences, Bejing 100101, China

[4] School of Natural and Environmental Sciences, Newcastle University, Newcastle upon Tyne, NE1 7RU

[5] School of Cellular and Molecular Medicine, University of Bristol, BS8 1TD, UK

[#] These authors contributed equally

*E-mail: chris.willis@bristol.ac.uk, matt.crump@bristol.ac.uk

1. **General Experimental Details** 3

2. **Isolation of Natural Products from Mutant Strains of *P. fluorescens***

NCIMB 10586 3

DesepoxyPA-B **4** 4

Pseudomonic acid C **3** 4

3. **Synthetic Procedures** 5-20

Preparation of protected monic acid **11** from PA-A **1** 5

6,7-*O*-Isopropylidenemonic acid C **S-2** 6

13-(*tert*-Butyldimethylsilyloxy)-6,7-*O*-isopropylidenemonic acid C **11** 6

Synthesis of hydroxy esters for coupling with acid **11** 7

Hydroxy ester **S-4** 7

General Procedure for conversion of acid **11** to esters **12**, **13** and **14** 8

C_7 Pseudomonic acid C **12** 9

C_8 Pseudomonic acid C **13** 9

C_{10} Pseudomonic acid C **14** 10

N-Methoxy-*N*-methyl 13-(*tert*-butyldimethylsilyloxy)-6,7-*O*-isopropylidenemonic amide C (**15**) 10
Addition of Grignard reagent to Weinreb amide **15** 11
Preparation of primary alcohol **S-6** 12
Preparation of ketones **17** and **18** 12
Reduction of carboxylic acid **11** 14
Preparation of alkyne **19** 14
Preparation of 9-azidononanoic acid **20** 15
Preparation of acid **S-10** 16
Deprotection of **S-10** to give **21** 17
Synthesis of **22** 18
Protection of tetrol **22** 18
Esterification of acid **23** to give ester **25** 19
Deprotection of silyl ether **25** to give 26 20
General procedure of whole-cell biotransformation with MupW strain 20
Oxidation to give C_7-desepoxy-PA-B **27** 21
General experimental procedures for feedings 21
Feeding desepoxy PA-B **4** and **27** to *ΔmupW* or *ΔmupH* of *P. fluorescens* mutant 21
4. **NMR Spectra** 23
5. **References** 31

1. General experimental details

All reactions were carried out using standard Schlenk syringe-septa techniques in flame dried glassware under a positive pressure of nitrogen in anhydrous solvents unless otherwise stated. Reagents and solvents were purchased from commercial suppliers and used without further purification unless reported. Anhydrous THF, Et_2O, and DCM were dried by passing through a modified Grubbs system of alumina columns and stored under nitrogen. Thin-layer chromatography TLC analysis was carried out on Merck silica gel 60 F_{254} analytical plates with a suitable solvent system and was visualized using UV radiation (254 & 366 nm) and/or developed with $KMnO_4$ / Δ. Flash column chromatography was performed using silica gel 60 (Fisher Scientific or Aldrich) and a suitable eluent. Infrared spectra were recorded on a Perkin-Elmer FT-IR spectrometer spectrum 2 with selected peaks of interest reported as absorption maxima (cm^{-1}). Mass spectrometry (MS) and High-resolution mass spectrometry (HRMS) were performed by the University of Bristol mass spectrometry service using electrospray ionisation (ESI) on a Bruker microOTOF II (TOF) or atmospheric pressure chemical ionisation (APCI) on a Thermo Scientific Orbitrap Elite (LC-Orbitrap). Optical rotation was measured on a Bellingham and Stanley Ltd. ADP220 polarimeter and is quoted in (° ml)(g dm)$^{-1}$. 1H and 13C NMR spectra were measured using Bruker and Jeol/Varian 400 MHz or Bruker Cryo 500/600 MHz, spectrometers at ambient temperature. Spectra were recorded in deuterochloroform referenced to residual $CHCl_3$ (^1H, 7.26 ppm; ^{13}C, 77.2 ppm), deuterated methanol referenced to residual MeOH (^1H, 4.87 ppm; ^{13}C, 49.0 ppm). Chemical shifts (δ) are quoted in parts per million (ppm) and coupling constants (*J*) are in hertz (Hz). The following abbreviations are used to describe multiplicity: s (singlet), d (doublet), t (triplet), q (quartet), p (pentet), sext (sextet), br. (broad). COSY, HMBC, and HSQC NMR spectra were routinely used to definitively assign the signals of ^1H and ^{13}C NMR spectra. For clarity, the numbering of atoms does not correspond to the compound names. LC-MS data were obtained on a Waters LC-MS system; Waters 2767 autosampler, Waters 515 HPLC Pump, Waters 2998 Photodiode Array Detector, Waters 2424 ELS detector, Waters Quattro mass spectrometer, with HPLC-grade H_2O and MeCN with 0.05% formic acid solvent system. Preparative HPLC purification was carried out using a Phenomenex Kinetex column (C18, 250 mm x 21.20 mm, 5 µm), at a flow rate of 16 mL min^{-1}.

2. Isolation of Natural Products from Mutant Strains of *P. fluorescens* NCIMB 10586 General Procedure.

The procedure was based literature methods. [1] *P. fluorescens* mutant strains were inoculated on LB-agar plates (1% Bacto tryptone, 0.5% yeast extract, 0.5% sodium chloride, 0.1% glucose, 1.5% agar) and incubated overnight at 30 °C. Seed medium was inoculated in a 50

mL Falcon tube with 10 mL of L-broth (1% Bacto tryptone, 0.5% yeast extract, 0.5% sodium chloride, 0.1% glucose) by picking a single colony from the L-agar plate and incubated overnight at 200 rpm at 30 °C. Production fermentation was inoculated with 5% of seed culture in 50 mL of L-broth with 4% w/v glucose in a 250 mL baffled flask. The culture was incubated at 22 °C at 200 rpm for 2 days and then centrifuged at 7000 rpm for 15 mins. The supernatant was extracted with EtOAc 3 times and the combined EtOAc extracts were evaporated *in vacuo* to give a crude extract, which was subjected to LC-MS analysis or further purification.

DesepoxyPA-B 4

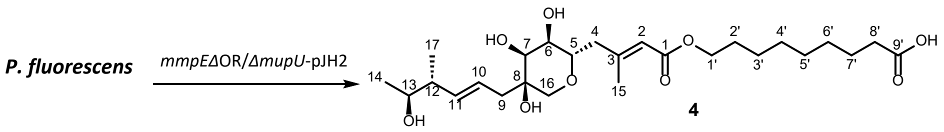

Fermentation of *P. fluorescens* mutant mmpEΔOR/ΔmupU-pJH$_2$ was conducted on 2.25 L scale as described in the general procedure. The crude extract was purified by column chromatography using an 100% EtOAc and a drop of AcOH (0.5 ml/1 L) to give desepoxyPA-B **4** (0.650 g/2.25 L) as a green oil. δ_H (400 MHz, CD_3OD) 5.73 (1H, br s, 2-H), 5.58 (1H, m, 10-H), 5.46 (1H, dd, *J* 15.6, 7.6, 11-H), 4.06 (2H, t, *J* 6.6, 1'-H$_2$), 3.71 (1H, d, *J* 2.8, 7-H), 3.64 (1H, m, 5-H), 3.61 (1H, m, 13-H), 3.43 (1H, d, *J* 10.8, 16-*H*H), 3.34- 3.32 (2H, m, 6-H and 16-H*H*), 2.65 (1H, d, *J* 14.2, 4-*H*H), 2.32 (2H, d, *J* 7.1, 9-H$_2$), 2.27 (2H, t, *J* 7.5, 8'-H$_2$), 2.20 – 2.10 (5H, m, 12-H, 4-H*H* and 15-H$_3$), 1.69 – 1.55 (4H, m, 2'-H$_2$ and 7'-H$_2$), 1.42 – 1.27 (8H, m, 3'-H$_2$, 4'-H$_2$, 5'-H$_2$, and 6'-H$_2$), 1.10 (3H, d, *J* 6.3, 14-H$_3$), 1.00 (3H, d, *J* 6.9, 17-H$_3$). δ_C (100 MHz, CD_3OD) 177.4 (C-9'), 168.1 (C-1), 158.8 (C-3), 137.1 (C-11), 125.6 (C-10), 118.0 (C-2), 73.8 (C-5), 72.5 (C-7), 71.8 (C-8), 70.5 (C-13), 69.8 (C-6) 69.4 (C-16), 64.5 (C-1'), 45.2 (C-12), 43.7 (C-4), 39.4 (C-9), 34.7 (C-8'), 30.0 (C-3', C-5', C-6' or C-7'), 29.9 (C-3', C-5', C-6' or C-7'), 29.5 (C-3', C-5', C-6' or C-7'), 29.1 (C-C2'), 26.8 (C-3', C-5', C-6' or C-7'), 25.8 (C-4'), 20.0 (C-14), 19.1 (C-15), 16.2 (C-17)Data in accord with the literature. [2]

Pseudomonic acid C 3

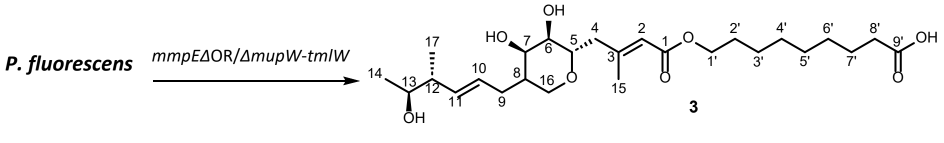

Fermentation of *P. fluorescens* mutant *mmpE*ΔOR/Δ*mupW-tmlW* was conducted on 2.25 L scale as described previously [1] and in the general procedure. The solvent was removed in *vacuo* and the crude product was purified by flash column chromatography (SiO_2, 0-6% MeOH in CH_2Cl_2) giving PA-C **3** (0.765 g/2.25L) as a green oil. δ_H(400 MHz, $CDCl_3$) 0.99 (3H, d, *J* 6.9, 14-H$_3$), 1.16 (3H, d, *J* 6.2, 17-H$_3$), 1.30-1.40 (8H, m, 3'-H$_2$, 4'-H$_2$, 5'-H$_2$, and 6'-H$_2$), 1.58-1.68 (4H, m, 2'-H$_2$ and 7'-H$_2$), 1.85 (1H, m, 8-H), 2.06-2.32 (4H, m, 4-*H*H, 9-H$_2$, 12-H), 2.22

(3H, s, 15-H_3), 2.34 (2H, t, *J* 7.3, 8'-H_2), 2.62 (1H, dd, *J* 14.5, 2.6, 4-H*H*), 3.47 (1H, dd, *J* 8.7, 2.7, 6-H), 3.51-3.59 (2H, m, 13-H, 16-*H*H), 3.74 (1H, m, 5-H), 3.80 (1H, dd, *J* 11.8, 3.0, 16-H*H*), 3.92 (1H, br t, *J* 3.2, 7-H), 4.09 (2H, t, *J* 6.2, 1'-H_2), 5.41 (1H, dd, *J* 15.4, 8.0, 11-H), 5.50 (1H, dt, *J* 15.4, 6.5, 10-H), 5.77 (1H, s, 2-H); δ_C(100 MHz, $CDCl_3$) 16.8 (C-17), 19.3 (C-15), 20.5 (C-14), 24.8 (C-5'), 26.1, 28.7, 29.0, 29.1 (C-3', C-4', C-6', C-7'), 29.2 (C-2'), 32.5 (C-9), 33.9 (C-8'), 42.1 (C-8), 43.2 (C-4), 44.9 (C-12), 64.0 (C-1'), 65.0 (C-16), 69.1 (C-6), 70.5 (C-7), 71.4 (C-13), 74.9 (C-5), 117.7 (C-2), 129.7 (C-10), 134.6 (C-11), 156.9 (C-3), 167.0 (C-1), 177.9 (C-9'). Spectroscopic data is accord with the literature.[3,4]

3. Synthetic Procedures

Preparation of protected monic acid 11 from PA-A 1

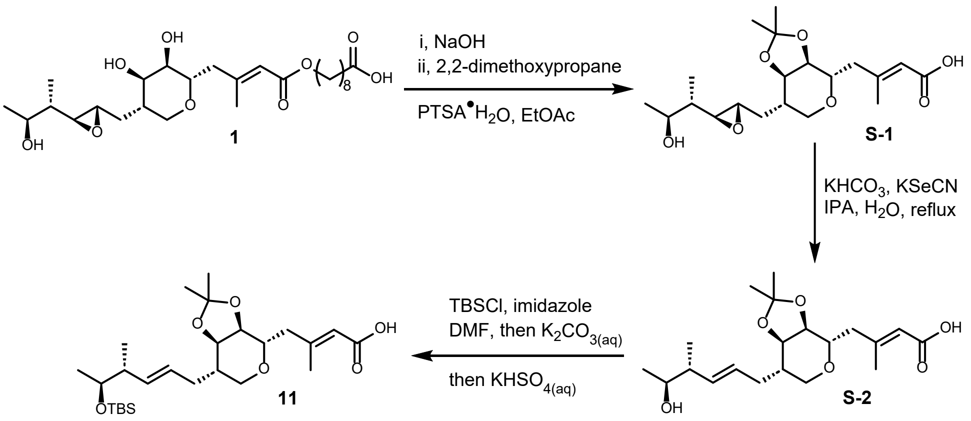

Pseudomonic acid A **1** (0.486 g, 0.98 mmol) was dissolved in 0.3 M NaOH (125 mL) and stirred for 24 hours at room temperature. The reaction mixture was concentrated to ~50 mL, acidified to ~pH 2 with 2 M HCl. The resulting solution was extracted with EtOAc (3 x 150 mL) then the combined organic layers were dried over $MgSO_4$, and the solvent was removed *in vacuo.* The crude material was used in the next step without purification.

The product (0.498 g, 1.445 mmol) from the above reaction was dissolved in EtOAc (4.3 mL) and 2,2-dimethoxypropane (4.3 mL). PTSA•H_2O (0.028 g, 0.144 mmol) was added, and then the reaction was stirred at room temperature for 1 h. Brine (20 mL) was added, and the aqueous phase was extracted with EtOAc (5 × 30 mL). The combined organic layers were dried over $MgSO_4$, filtered and concentrated *in vacuo*. The crude product was purified by flash column chromatography (SiO_2, 100% EtOAc) giving acid **S-1** (0.500 g, 90%) as a colourless oil. δ_H(400 MHz, $CDCl_3$) 0.94 (3H, d, *J* 7.0, 17-H_3), 1.20 (3H, d, *J* 6.3, 14-H_3), 1.32 (1H, m, 12-H), 1.35 (3H, s, 1'-CH_3), 1.49 (3H, s, 1'-CH_3), 1.64-1.79 (2H, m, 9-H_2), 2.17 (1H, m, 8-H), 2.18 (3H, d, *J* 0.9, 15-H_3), 2.23 (1H, dd, *J* 14.7, 9.2, 4-*H*H), 2.50 (1H, dd, *J* 14.7, 2.7, 4-H*H*), 2.67 (1H, dd, *J* 8.1, 2.2, 11-H), 2.84 (1H, ddd, *J* 7.1, 5.0, 2.2, 10-H), 3.45 (1H, td, *J* 9.2, 2.7, 5-H), 3.67 (1H, dd, *J* 11.7, 1.8, 16-*H*H), 3.72-3.77 (2H, m, 6-H and 16-H*H*), 3.82 (1H, *app.* p, *J* 6.4, 13-H), 4.14 (1H, dd, *J* 4.9, 2.4, 7-H), 5.75 (1H, s, 2-H); δ_C(100 MHz, $CDCl_3$) 12.9 (C-17), 19.5

(C-15), 20.7 (C-14), 26.4 (1'-CH_3), 28.4(1'-CH_3), 33.9 (C-9), 35.2 (C-8), 44.3 (C-4), 55.7 (C-10), 61.6 (C-11), 67.1 (C-16), 71.4 (C-13), 74.3 (C-6), 75.7 (C-7), 76.4 (C-5), 109.0 (C-1'), 117.3 (C-2), 159.1 (C-3), 171.3 (C-1). Spectroscopic data is accord with the literature. [5]

6,7-*O*-Isopropylidenemonic acid C S-2

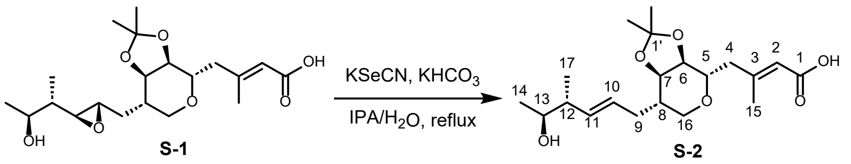

Acid **S-1** (0.022 g, 0.056 mmol) was dissolved in 50% $MeOH_{(aq)}$ (0.2 mL) and then $KHCO_3$ (0.005 g) was added. The solution was concentrated *in vacuo* and then re-dissolved in IPA (0.76 mL) and H_2O (0.08 mL). KSeCN (0.024 g, 0.168 mmol) was added, and then the reaction was heated to reflux for 2 days. After cooling back to room temperature, 2 M $HCl_{(aq)}$ was added to pH 2. The aqueous phase was extracted with EtOAc (4 × 20 mL). The combined organic layers were dried over $MgSO_4$, filtered and concentrated *in vacuo*. The crude product was purified by flash column chromatography (SiO_2, 1-10% MeOH in CH_2Cl_2) giving acid **S-2** (0.012 g, 60%) as a colourless oil. δ_H(400 MHz, $CDCl_3$) 1.00 (3H, d, *J* 6.9, 17-H_3), 1.16 (3H, d, *J* 6.3, 14-H_3), 1.35 (3H, s, 1'-CH_3), 1.50 (3H, s, 1'-CH_3), 2.01 (1H, m, 8-H), 2.07-2.30 (4H, m, 4-*H*H, 9-H_2, 12-H), 2.20 (3H, s, 15-H_3), 2.52 (1H, dd, *J* 15.2, 2.1, 4-H*H*), 3.44 (1H, td, *J* 9.2, 2.9, 5-H), 3.57 (1H, *app.* p., *J* 6.3, 13-H), 3.62 (1H, dd, *J* 11.6, 1.5, 16-*H*H), 3.65-3.72 (2H, m, 6-H, 16-H*H*), 4.12 (1H, dd, *J* 4.9, 2.2, 7-H), 5.43 (1H, dd, *J* 15.4, 8.0, 11-H), 5.53 (1H, dt, *J* 15.4, 6.8, 10-H), 5.78 (1H, br. s, 2-H); δ_C(100 MHz, $CDCl_3$) 16.7 (C-17), 19.5 (C-15), 20.5 (C-14), 26.4 and 28.5 ($O_2C(CH_3)_2$), 34.3 (C-9), 36.8 (C-8), 44.3 (C-4), 44.9 (C-12), 66.6 (C-16), 71.2 (C-13), 74.3 (C-6), 75.7 (C-7), 76.5 (C-5), 108.8 (C-1'), 117.3 (C-2), 129.4 (C-11), 135.0 (C-10), 159.2 (C-3), 171.4 (C-1). Spectroscopic data is accord with the literature. [Error! Bookmark not defined.]

13-(*tert*-Butyldimethylsilyloxy)-6,7-*O*-isopropylidenemonic acid C 11

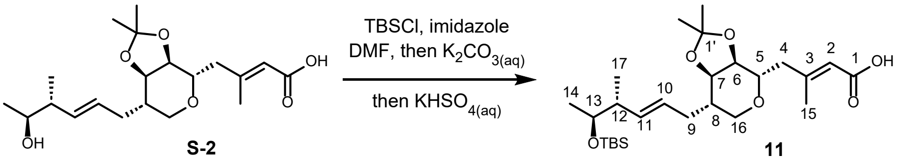

Acid **S-2** (0.531 g, 1.440 mmol) was dissolved in anhydrous DMF (4.3 mL) under an atmosphere of nitrogen, and then imidazole (0.431 g, 6.336 mmol) was added. TBSCl (0.478 g, 3.168 mmol) was added, and then the reaction was stirred at room temperature for 16 h. H_2O (10 mL) and Et_2O (30 mL) were added, and the organic phase was washing with H_2O (2

× 5 mL). The organic layer was dried over $MgSO_4$, filtered and concentrated *in vacuo*. The residue was dissolved in MeOH (10.5 mL) and THF (3.5 mL). K_2CO_3 (0.348 g) in H_2O (3.5 mL) was added dropwise and stirred at room temperature for 1 h. After concentrating *in vacuo*, brine (20 mL) was added. 10% $KHSO_{4(aq)}$ was added to pH 4. The aqueous phase was extracted with EtOAc (5 × 50 mL). The combined organic layers were dried over MgSO4, filtered and concentrated *in vacuo*. The crude product was purified by flash column chromatography (SiO_2, 1% MeOH in CH_2Cl_2) giving acid **11** (0.647 g, 93%) as a colourless oil. $[\alpha]_D^{20}$ –20 (*c* 1.0, $CHCl_3$); 2957, 2929, 2886, 2857, 1694, 1642; δ_H(400 MHz, $CDCl_3$) 0.02 (6H, s, $Si(CH_3)_2$), 0.87 (9H, s, $SiC(CH_3)_3$), 0.96 (3H, d, *J* 6.9, 17-H_3), 1.02 (3H, d, *J* 6.2, 14-H_3), 1.35 (3H, s, 1'-CH_3), 1.50 (3H, s, 1'-CH_3), 1.98 (1H, m, 8-H), 2.11-2.28 (4H, m, 4-*H*H, 9-H_2, 12-H), 2.20 (3H, s, 15-H_3), 2.52 (1H, dd, *J* 15.0, 1.6, 4-H*H*), 3.42 (1H, td, *J* 9.3, 2.8, 5-H), 3.61-3.71 (4H, m, 6-H, 13-H, 16-H_2), 4.13 (1H, dd, *J* 4.9, 2.1, 7-H), 5.37 (1H, dt, *J* 15.4, 6.3, 10-H), 5.45 (1H, dd, *J* 15.4, 7.2, 11-H), 5.77 (1H, br. s, 2-H); δ_C(100 MHz, $CDCl_3$) –4.7 and –4.2 ($Si(CH_3)_2$), 16.1 (C-17), 18.2 (SiC), 19.5 (C-15), 20.7 (C-14), 26.0 ($SiC(CH_3)_3$), 26.4 and 28.5 ($O_2C(CH_3)_2$), 34.3 (C-8), 36.9 (C-9), 44.4 (C-4 and C-12), 66.8 (C-16), 71.9 (C-13), 74.2 (C-6), 75.5 (C-7), 76.6 (C-5), 108.8 (C-1'), 117.2 (C-2), 127.3 (C-10), 135.8 (C-11), 159.7 (C-3), 171.7 (C-1); Found (ESI): 483.3149 $[M+H]^+$, (required $C_{24}H_{41}O_8$ 483.3136); Found (ESI): 505.2952 $[M+Na]^+$, (required $C_{24}H_{40}O_8Na$ 505.2956).

Synthesis of hydroxy esters for coupling with acid 11

Hydroxy ester S-4

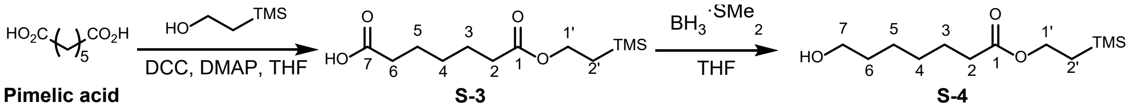

Pimelic acid (2.794 g, 17.442 mmol) was dissolved in anhydrous THF (26.4 mL) under an atmosphere of nitrogen. 2-(Trimethylsilyl)ethanol (0.5 mL, 3.488 mmol) and DMAP (0.043 g, 0.349 mmol) were added separately followed by the addition of DCC (0.669 g, 3.488 mmol) in anhydrous THF (4.4 mL). The reaction was stirred for 16 h, filtered through a plug of Celite® and concentrated *in vacuo*. The crude product was purified by flash column chromatography (SiO_2, 20% EtOAc in petroleum ether 40-60 °C) giving acid **S-3** (0.737 g, 81%) as a colourless oil. ν_{max}/cm^{-1} 3033, 2952, 2868, 1732, 1708; δ_H(400 MHz, $CDCl_3$) 0.03-0.05 (9H, m, $Si(CH_3)_3$), 0.94-1.01 (2H, m, 2'-H_2), 1.33-1.43 (2H, m, 4-H_2), 1.59-1.70 (4H, m, 3-H_2, 5-H_2), 2.29 (2H, td, *J* 7.6, 1.5, 2-H_2), 2.35 (2H, td, *J* 7.5, 2.1, 6-H_2), 4.12-4.19 (2H, m, 1'-H_2); δ_C(100 MHz, $CDCl_3$) -1.4 ($Si(CH_3)_3$), 17.5 (C-2'), 24.4, 24.7 (C-3, C-5), 28.7 (C-4), 33.9 (C-6), 34.4 (C-2), 62.7 (C-1'), 173.9 (C-1), 179.6 (C-7). Spectroscopic data is accord with the literature. [6]

Acid **S-3** (0.737 g, 2.829 mmol) was dissolved in anhydrous THF (2.83 mL) and was cooled to 0 °C under an atmosphere of nitrogen. BH_3•SMe_2 (2 M, 2.83 mL, 5.657 mmol) was added dropwise, and the reaction was stirred at the same temperature for 1 h followed by the addition of H_2O (10 mL) dropwise. The aqueous phase was extracted with Et_2O (3 × 20 mL). The combined organic layers were dried over $MgSO_4$, filtered and concentrated *in vacuo*. The crude product was purified by flash column chromatography (SiO_2, 20% EtOAc in petroleum ether 40-60 °C) giving alcohol **S-4** (0.592 g, 85%) as a colourless oil. ν_{max}/cm^{-1} 3417, 2935, 2860, 1732; δ_H(400 MHz, $CDCl_3$) 0.03 (9H, s, $Si(CH_3)_3$), 0.94-1.01 (2H, m, 2'-H_2), 1.29-1.43 (4H, m, 4-H_2, 5-H_2), 1.51-1.59 (2H, m, 6-H_2), 1.60-1.67 (2H, m, 3-H_2), 2.27 (2H, t, *J* 7.4, 2-H_2), 3.63 (2H, t, *J* 6.6, 7-H_2), 4.12-4.18 (2H, m, 1'-H_2); δ_C(100 MHz, $CDCl_3$) -1.4 ($Si(CH_3)_3$), 17.5 (C-2'), 25.0 (C-3), 25.5, 29.0 (C-4, C-5), 32.7 (C-6), 34.6 (C-2), 62.6 (C-1'), 63.0 (C-7), 174.1 (C-1); Found (ESI): 269.1545 $[M+Na]^+$, (required $C_{12}H_{26}O_3SiNa$ 269.1543).

General Procedure for conversion of acid 11 to esters 12, 13 and 14

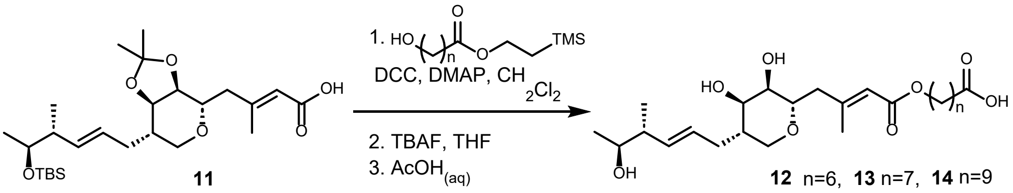

Acid **11** (1 eq.) was dissolved in anhydrous CH_2Cl_2 (5.35 mL/mmol of the substrate), and then DCC (2.2 eq.) and DMAP (0.22 eq.) were added under an atmosphere of nitrogen. The primary alcohol (2.4 eq.) in anhydrous CH_2Cl_2 (5.35 mL/mmol of the substrate) was added, and then the reaction was stirred for 18 h. The mixture was filtered through a plug of Celite® and concentrated *in vacuo*. The crude product was purified by flash column chromatography (SiO_2, 5% EtOAc in petroleum ether 40-60 °C) to give ester. The ester was dissolved in anhydrous THF (20.9 mL/mmol of the substrate), and then TBAF (3.76 eq.) was added dropwise under an atmosphere of nitrogen. The reaction was stirred for 16 h, and then $HCl_{(aq)}$ (2 M) was added to adjust the pH to 4. The aqueous phase was extracted with EtOAc (3 times). The combined organic layers were dried over $MgSO_4$, filtered and concentrated *in vacuo*. The crude product was dissolved in $AcOH_{(aq)}$ (80%, 7.5 mL/mmol of the substrate), stirred for 16 h, and concentrated *in vacuo*. The crude product was purified by flash column chromatography giving acid **12, 13** or **14**.

C_7 Pseudomonic acid C 12

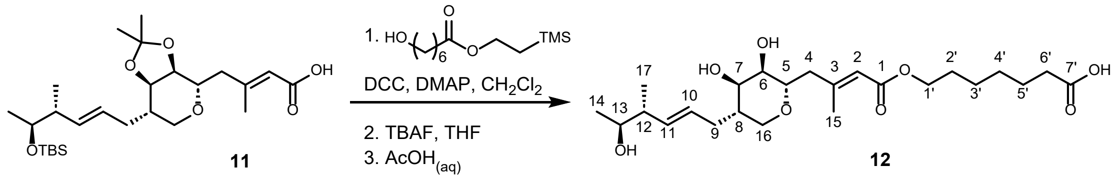

Acid **11** (0.090 g, 0.187 mmol) was used to synthesise acid **12** (0.029 g, 34% over 3 steps) as a colourless oil *via* the above procedure. $[\alpha]_D^{24}$ +37 (*c* 0.27, $CHCl_3$); ν_{max}/cm^{-1} 3417, 2926, 2868, 1710, 1646; δ_H(500 MHz, $CDCl_3$) 0.99 (3H, d, *J* 6.9, 17-H_3), 1.16 (3H, d, *J* 6.3, 14-H_3), 1.35-1.45 (4H, m, 3'-H_2 and 4'-H_2), 1.60-1.69 (4H, m, 2'-H_2 and 5'-H_2), 1.85 (1H, m, 8-H), 2.05-2.13 (1H, m, 12-H), 2.13-2.26 (2H, m, 9-H_2), 2.21 (3H, br s, 15-H_3), 2.29 (1H, dd, *J* 14.6, 8.8, 4-*H*H), 2.34 (2H, t, *J* 7.3, 6'-H_2), 2.62 (1H, dd, *J* 14.6, 1.9, 4-H*H*), 3.47 (1H, dd, *J* 8.8, 2.9, 6-H), 3.51-3.59 (2H, m, 13-H, 16-*H*H), 3.74 (1H, td, *J* 8.8, 3.0, 5-H), 3.80 (1H, dd, *J* 11.6, 2.7, 16-H*H*), 3.91 (1H, m, 7-H), 4.04-4.14 (2H, m, 1'-H_2), 5.41 (1H, dd, *J* 15.5, 8.1, 11-H), 5.50 (1H, dt, *J* 15.5, 6.7, 10-H), 5.77 (1H, s, 2-H); δ_C(125 MHz, $CDCl_3$) 16.9 (C-17), 19.3 (C-15), 20.5 (C-14), 24.7 (C-5'), 26.0 (C-4'), 28.4 (C-2'), 28.7 (C-3'), 32.4 (C-9), 33.8 (C-6'), 41.9 (C-8), 43.1 (C-4), 44.9 (C-12), 63.9 (C-1'), 65.0 (C-16), 69.0 (C-6), 70.5 (C-7), 71.4 (C-13), 74.9 (C-5), 117.8 (C-2), 129.7 (C-10), 134.6 (C-11), 156.9 (C-3), 166.9 (C-1), 177.6 (C-7'); Found (ESI): 457.2791 $[M+H]^+$, (required $C_{24}H_{41}O_8$ 457.2796); Found (ESI): 479.2613 $[M+Na]^+$, (required $C_{24}H_{40}O_8Na$ 479.2615).

C_8 Pseudomonic acid 13

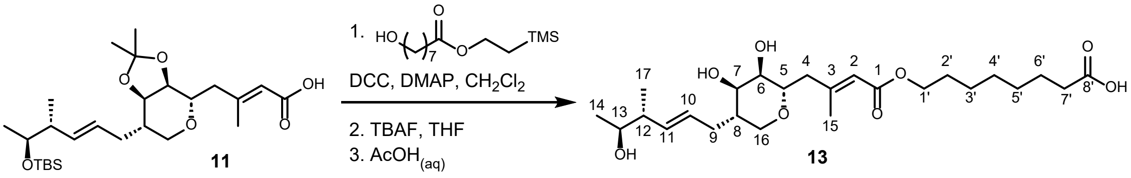

Acid **11** (0.088 g, 0.182 mmol) was used to synthesise acid **13** (0.029 g, 34% over 3 steps) as a colourless oil *via* the above general procedure. $[\alpha]_D^{20}$ –22 (*c* 0.45, MeOH); ν_{max}/cm^{-1} 3418, 2930, 2870, 1712, 1646, 1447; δ_H(400 MHz, CD_3OD) 0.99 (3H, d, *J* 7.0, 17-H_3), 1.09 (3H, d, *J* 6.4, 14-H_3), 1.33-1.43 (6H, m, 3'-H_2, 4'-H_2 and 5'-H_2), 1.56-1.68 (4H, m, 2'-H_2 and 6'-H_2), 1.76 (1H, m, 8-H), 2.12-2.23 (4H, m, 4-*H*H, 9-H_2 and 12-H), 2.18 (3H, s, 15-H_3), 2.28 (2H, t, *J* 7.4, 7'-H_2), 2.65 (1H, br d, *J* 14.2, 4-H*H*), 3.35 (1H, m, 6-H), 3.51 (1H, br d, *J* 11.4, 16-*H*H), 3.60 (1H, qd, *J* 6.4, 5.2, 13-H), 3.72 (1H, td, *J* 9.4, 2.5, 5-H), 3.77 (1H, dd, *J* 11.4, 2.8, 16-H*H*), 3.83 (1H, br t, *J* 3.2, 7-H), 4.07 (2H, t, *J* 6.6, 1'-H_2), 5.38-5.48 (2H, m, 10-H and 11-H), 5.74 (1H, s, 2-H); δ_C(100 MHz, CD_3OD) 16.7 (C-17), 19.3 (C-15), 20.3 (C-14), 26.0 (C-6'), 27.0 (C-2'), 29.8, 30.0, 30.1 (C-3', C-4', C-5'), 33.7 (C-9), 34.9 (C-7'), 43.7, 44.1 (C-4, C-8), 45.3 (C-12), 64.8 (C-1'), 65.7 (C-16), 69.9 (C-6), 71.5 (C-7), 72.1 (C-13), 76.1 (C-5), 118.2 (C-2), 129.7

(C-10), 135.8 (C-11), 159.1 (C-3), 168.4 (C-1), 177.7 (C-8'); Found (ESI): 493.2766 [M+Na]$^+$, (required $C_{25}H_{42}NaO_8$ 493.2772).

C_{10} Pseudomonic acid C 14

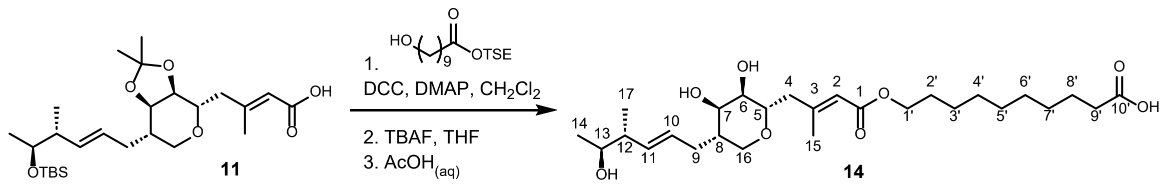

Acid **11** (0.078 g, 0.161 mmol) was used to synthesise acid **14** (0.020 g, 25% over 3 steps) as a colourless oil *via* the above general procedure. $[\alpha]_D^{24}$ +36 (*c* 0.224, $CHCl_3$); ν_{max}/cm^{-1} 3417, 2924, 2855, 1710, 1646; δ_H(500 MHz, $CDCl_3$) 0.99 (3H, d, *J* 6.9, 17-H_3), 1.16 (3H, d, *J* 6.2, 14-H_3), 1.27-1.40 (10H, m, 3'-H_2, 4'-H_2, 5'-H_2, 6'-H_2 and 7'-H_2), 1.58-1.67 (4H, m, 2'-H_2 and 8'-H_2), 1.85 (1H, m, 8-H), 2.10 (1H, m, 12-H), 2.12-2.26 (2H, m, 9-H_2), 2.21 (3H, d, *J* 1.2, 15-H_3), 2.28 (1H, dd, *J* 14.6, 9.0, 4-*H*H), 2.33 (2H, t, *J* 7.4, 9'-H_2), 2.62 (1H, dd, *J* 14.6, 2.1, 4-H*H*), 3.45 (1H, dd, *J* 8.9, 3.1, 6-H), 3.52 (1H, m, 16-*H*H), 3.56 (1H, m, 13-H), 3.72 (1H, td, *J* 9.0, 3.1, 5-H), 3.80 (1H, dd, *J* 11.7, 2.9, 16-H*H*), 3.91 (1H, t, *J* 3.1, 7-H), 4.08 (2H, t, *J* 6.5, 1'-H_2), 5.41 (1H, dd, *J* 15.4, 8.1, 11-H), 5.50 (1H, dt, *J* 15.4, 6.8, 10-H), 5.76 (1H,d, *J* 0.9, 2-H); δ_C(125 MHz, $CDCl_3$) 16.8 (C-17), 19.3 (C-15), 20.5 (C-14), 24.8 (C-8'), 26.1, 29.00, 29.03, 29.1, 29.2 (C-3', C-4', C-5', C-6', C-7'), 28.7 (C-2'), 32.5 (C-9), 33.9 (C-9'), 42.1 (C-8), 43.2 (C-4), 44.9 (C-12), 64.0 (C-1'), 65.0 (C-16), 69.1 (C-6), 70.5 (C-7), 71.4 (C-13), 74.9 (C-5), 117.7 (C-2), 129.7 (C-10), 134.6 (C-11), 156.9 (C-3), 167.0 (C-1), 177.9 (C-10); Found (ESI): 499.3254 [M+H]$^+$, (required $C_{27}H_{47}O_8$ 499.3265); Found (ESI): 521.3076 [M+Na]$^+$, (required $C_{27}H_{46}O_8Na$ 521.3085).

***N*-Methoxy-*N*-methyl 13-(*tert*-butyldimethylsilyloxy)-6,7-*O*-isopropylidenemonic amide C (15)**

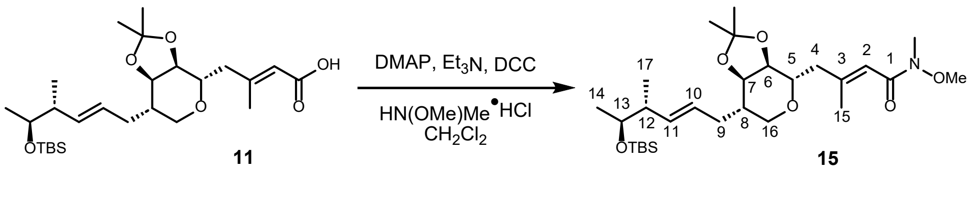

Acid **11** (0.218 g, 0.452 mmol) was dissolved in anhydrous CH_2Cl_2 (5.2 mL) and then cooled to 0 °C under an atmosphere of nitrogen. Et_3N (0.139 mL, 0.995 mmol), DCC (0.205 g, 0.995 mmol) and DMAP (0.012 g, 0.099 mmol) were added, and then the mixture was stirred at room temperature for 0.5 h. After cooling back to 0 °C, HN(OMe)Me•HCl (0.097 g, 0.995 mmol) was added. The reaction was stirred at room temperature for 16 h and then filtered and concentrated *in vacuo*. The crude product was purified by flash column chromatography (SiO_2,

10-30% EtOAc in petroleum ether 40-60 °C) giving Weinreb amide **15** (0.180 g, 76%) as a colourless oil. $[\alpha]_D^{21}$ −140 (*c* 1.0, $CHCl_3$); ν_{max}/cm^{-1} 2957, 2928, 2857, 1657, 1637; δ_H(400 MHz, $CDCl_3$) 0.03 (6H, s, $Si(CH_3)_2$), 0.88 (9H, s, $SiC(CH_3)_3$), 0.96 (3H, d, *J* 6.9, 17-H_3), 1.02 (3H, d, *J* 6.3, 14-H_3), 1.35 and 1.50 (each 3H, each s, $O_2C(CH_3)_2$), 1.98 (1H, m, 8-H), 2.11-2.27 (4H, m, 4-*H*H, 9-H_2 and 12-H), 2.16 (3H, s, 15-H_3), 2.50 (1H, br. d, *J* 14.6, 4-H*H*), 3.20 (3H, s, NCH_3), 3.42 (1H, td, *J* 9.3, 2.6, 5-H), 3.59-3.72 (7H, m, 6-H, 13-H, 16-H_2), 3.67 (3H, s, OCH_3), 4.13 (1H, dd, *J* 4.8, 2.0, 7-H), 5.38 (1H, dt, *J* 15.4, 6.2, 10-H), 5.46 (1H, dd, *J* 15.4, 7.0, 11-H), 6.20 (1H, br. s, 2-H); δ_C(100 MHz, $CDCl_3$) −4.7 and −4.2 ($Si(CH_3)_2$), 16.1 (C-17), 18.3 (SiC), 19.2 (C-15), 20.7 (C-14), 26.0 ($SiC(CH_3)_3$), 26.5 and 28.5 ($O_2C(CH_3)_2$), 34.3 (C-9), 37.0 (C-8), 44.3 and 44.4 (C-4, C-12), 61.5 (OCH_3), 66.8 (C-16), 71.9 (C-13), 74.3 (C-6), 75.6 (C-7), 76.8 (C-5), 108.7 ($O_2C(CH_3)_2$), 116.3 (C-2), 127.4 (C-10), 135.7 (C-11), 153.0 (C-3, assigned by HMBC), 168.1 (C-1, assigned by HMBC); Found (ESI): 526.3554 $[M+H]^+$, (required $C_{28}H_{52}NO_6Si$ 526.3558); 548.3367 $[M+Na]^+$, (required $C_{28}H_{51}NNaO_6Si$ 548.3378).

Addition of Grignard reagent to Weinreb amide 15

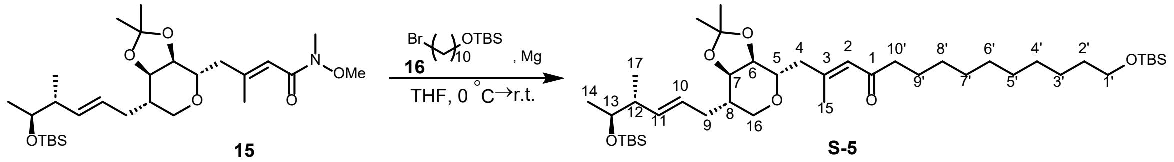

Bromide **16** (0.133 g, 0.378 mmol) and magnesium turnings (0.018 g, 0.756 mmol) was mixed with anhydrous THF (0.77 mL) under an atmosphere of nitrogen. The mixture was heated by hot air gun to reflux and then I_2 (catalytic amount) was added. The mixture was stirred for further 2 h to form a cloudy grey solution. The mixture was added to a solution of amide **15** (0.069 g, 0.130 mmol) at 0 °C and the reaction was stirred at the same temperature for 15 min and then at room temperature for 3 h. Saturated $NH_4Cl_{(aq)}$ (5 mL) was added, and the aqueous phase was extracted with EtOAc (3 × 20 mL). The combined organic layers were dried over $MgSO_4$, filtered and concentrated *in vacuo*. The crude product was purified by flash column chromatography (SiO_2, 4-6% EtOAc in petroleum ether 40-60 °C) giving ketone **S-5** (0.089 g, 93%) as a colourless oil. $[\alpha]_D^{22}$ −2 (*c* 1.0, $CHCl_3$); ν_{max}/cm^{-1} 2954, 2927, 2856, 1690, 1620; δ_H(400 MHz, $CDCl_3$) 0.03 and 0.04 (each 3H, each s, $Si(CH_3)_2$), 0.04 (6H, s, $Si(CH_3)_2$), 0.88 and 0.89 (each 9H, each s, 2 × $SiC(CH_3)_3$) 0.96 (3H, d, *J* 6.9, 17-H_3), 1.03 (3H, d, *J* 6.2, 14-H_3), 1.24-1.32 (12H, m, 3'-H_2, 4'-H_2, 5'-H_2, 6'-H_2, 7'-H_2 and 8'-H_2), 1.35 and 1.50 (each 3H, each s, $C(CH_3)_2$), 1.46-1.54 (2H, m, 2'-H_2), 1.54-1.62 (2H, m, 9'-H_2), 1.98 (1H, m, 8-H), 2.12-2.26 (4H, m, 4-*H*H, 9-H_2 and 12-H), 2.16 (3H, s, 15-H_3), 2.41 (2H, t, *J* 7.3, 10'-H_2), 2.48 (1H, br d, *J* 14.4, 4-H*H*), 3.41 (1H, td, *J* 9.2, 2.6, 5-H), 3.59 (2H, t, *J* 6.7, 1'-H_2), 3.64-3.71 (4H, m, 6-H, 13-H and 16-H_2), 4.13 (1H, dd, *J* 4.8, 2.1, 7-H), 5.39 (1H, dt, *J* 15.4, 6.2, 10-H), 5.46 (1H,

dd, J 15.4, 7.1, 11-H), 6.12 (1H, s, 2-H); δ_C(100 MHz, $CDCl_3$) –5.1 (1'-OSi$(CH_3)_2$), –4.7 and –4.2 (13-OSi$(CH_3)_2$), 16.2 (C-17), 18.2 and 18.5 (2 × SiC), 19.6 (C-15), 20.8 (C-14), 24.3 (C-9'), 25.9 (CH_2), 26.0, 26.1 (2 × SiC(CH_3)$_3$), 26.5 (CH_3), 28.5 (CH_3), 29.5 (CH_2), 29.6 (CH_2), 29.6 (CH_2), 29.6 (CH_2), 29.7 (CH_2), 33.0 (C-2'), 34.3 (C-9), 36.9 (C-8), 44.4, 44.5, 44.6 (C-4, C-12, C-10'), 63.5 (C-1'), 66.9 (C-16), 71.9 (C-13), 74.3 (C-6), 75.5 (C-7), 76.7 (C-5), 108.7 ($O_2C(CH_3)_2$), 125.3 (C-2), 127.3 (C-10), 135.7 (C-11), 154.6 (C-3), 201.5 (C-1); Found (ESI): 759.5391 $[M+Na]^+$, (required $C_{42}H_{80}O_6Si_2Na$ 759.5386).

Preparation of primary alcohol S-6

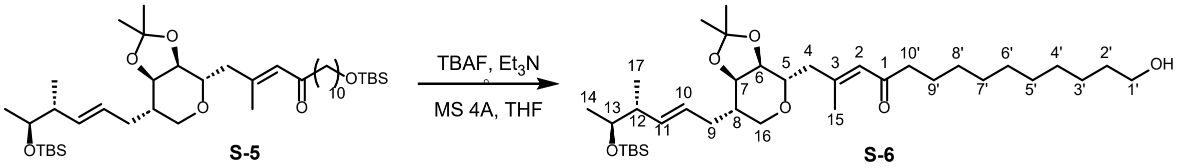

Silyl ether **S-5** (0.080 g, 0.108 mmol) was dissolved in anhydrous THF (1.2 mL) under an atmosphere of nitrogen. Molecular sieves 4Å (0.024 g) and Et_3N (0.072 mL) were added separately and then TBAF (1 M, 0.108 mL, 0.108 mmol) was added dropwise. The reaction was stirred at room temperature for 1 h and then concentrated *in vacuo*. The crude product was purified by flash column chromatography (SiO_2, 10-20% EtOAc in petroleum ether 40-60 °C) giving alcohol **S-6** (0.064 g, 95%) as a colourless oil. $[\alpha]_D^{21}$ –8 (c 1.0, $CHCl_3$); ν_{max}/cm^{-1} 3462, 2927, 2856, 1688, 1619; δ_H(500 MHz, $CDCl_3$) 0.02, 0.03 (each 3H, each s, Si$(CH_3)_2$), 0.88 (9H, s, SiC$(CH_3)_3$), 0.96 (3H, d, J 6.8, 17-H_3), 1.03 (3H, d, J 6.1, 14-H_3), 1.24-1.30 (12H, m, 3'-H_2, 4'-H_2, 5'-H_2, 6'-H_2, 7'-H_2 and 8'-H_2) , 1.35 (3H, s, CH_3), 1.50 (3H, s, CH_3), 1.51-1.58 (4H, m, 2'-H_2 and 9'-H_2), 1.98 (1H, m, 8-H), 2.12-2.27 (4H, m, 4-*H*H, 9-H_2 and 12-H), 2.16 (3H, s, 15-H_3), 2.41 (2H, t, J 7.5, 10'-H_2), 2.48 (1H, d, J 14.4, 4-H*H*), 3.20 (1H, s, OH), 3.41 (1H, td, J 9.2, 2.5, 5-H), 3.63 (2H, t, J 6.6, 1'-H_2), 3.65-3.69 (4H, m, 6-H, 13-H and 16-H_2), 4.14 (1H, m, 7-H), 5.38 (1H, dt, J 15.4, 6.5, 10-H), 5.45 (1H, dd, J 15.4, 7.4, 11-H), 6.12 (1H, s, 2-H); δ_C(125 MHz, $CDCl_3$) –4.7 and –4.2 (Si$(CH_3)_2$), 16.2 (C-17), 18.3 (SiC), 19.6 (C-15), 20.8 (C-14), 24.3 (C-9'), 25.9 (CH_2), 26.0 (SiC(CH_3)$_3$), 26.5 (CH_3), 28.5 (CH_3), 29.4 (CH_2), 29.5 (CH_2), 29.6 (CH_2), 29.6 (CH_2), 29.7 (CH_2), 33.0 (C-2'), 34.3 (C-9), 37.0 (C-8), 44.4, 44.5, 44.6 (C-10', C-4, C-12), 63.2 (C-1'), 66.9 (C-16), 71.9 (C-13), 74.3 (C-6), 75.5 (C-7), 76.7 (C-5), 108.8 ($O_2C(CH_3)_2$), 125.3 (C-2), 127.3 (C-10), 135.7 (C-11), 154.6 (C-3), 201.5 (C-1); Found (ESI): 645.4515 $[M+Na]^+$, (required $C_{36}H_{66}O_6SiNa$ 645.4521).

Preparation of ketones 17 and 18

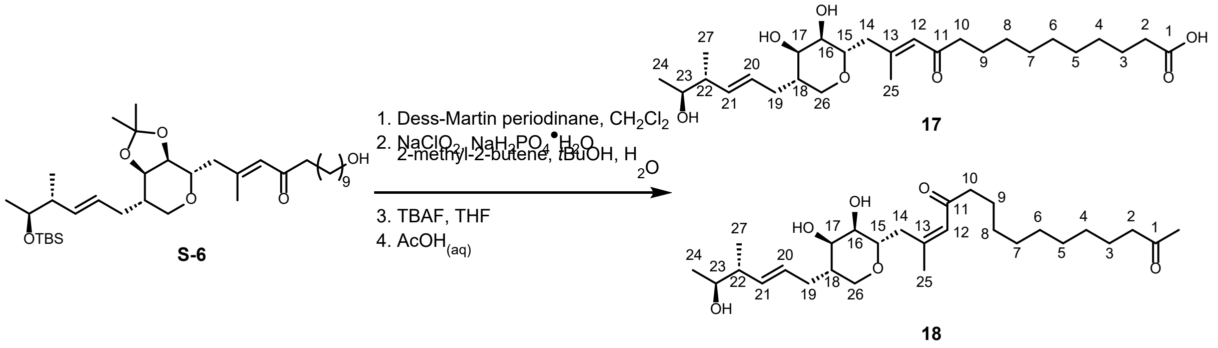

Alcohol **S-6** (0.082 g, 0.131 mmol) was dissolved in anhydrous CH_2Cl_2 (0.73 mL) and then cooled to 0 °C under an atmosphere of nitrogen. Dess-Martin periodinane (15% w/w, 0.472 mL) was added dropwise, and then the reaction was stirred at room temperature for 1.5 h. Saturated $NH_4Cl_{(aq)}$ (5 mL) was added, and then the aqueous phase was extracted with CH_2Cl_2 (3 × 20 mL). The combined organic layers were dried over $MgSO_4$, filtered and concentrated *in vacuo*. The crude aldehyde was used in next step without further purification. The crude aldehyde was dissolved in *t*BuOH (0.59 mL) and 2-methyl-2-butene (0.144 mL). A mixture of $NaClO_2$ (0.148 g, 1.308 mmol) and NaH_2PO_4•H_2O (0.181 g, 1.308 mmol) in H_2O (1.46 mL) was added dropwise. The solution was stirred vigorously for 1 h, and then EtOAc (5 mL) and H_2O (5 mL) were added. The aqueous phase was extracted with EtOAc (5 × 30 mL). The combined organic layers were dried over $MgSO_4$, filtered and concentrated *in vacuo*. The crude acid was used in the next step without further purification. The acid was dissolved in anhydrous THF (3.7 mL) under an atmosphere of nitrogen. TBAF (1.0 M, 0.262 mL, 0.262 mmol) was added dropwise, and then the reaction was stirred at room temperature for 16 h. H_2O (5 mL) was added, and then $HCl_{(aq)}$ (2 M) was added to pH 4. The aqueous phase was extracted with EtOAc (5 × 30 mL). The combined organic layers were dried over $MgSO_4$, filtered through a plug of Celite® and concentrated *in vacuo*. The crude acid was used in the next steps without further purification. The acid was dissolved in $AcOH_{(aq)}$ (80%, 1.3 mL). The reaction was stirred at room temperature for 16 h and then concentrated *in vacuo*. The crude product was purified by flash column chromatography (SiO_2, 1% MeOH in CH_2Cl_2) giving an inseparable mixture of acid **17** and **18** (12*E*:12*Z*::2:1, 0.014 g, 27% over 4 steps) as a colourless oil. ν_{max}/cm^{-1} 3420, 2925, 2855, 1725, 1713; *E*-isomer: δ_H(500 MHz, $CDCl_3$) 0.99 (3H, d, *J* 6.9, 27-H_3), 1.17 (3H, d, *J* 6.2, 24-H_3), 1.24-1.36 (10H, m, 4-H_2, 5-H_2, 6-H_2, 7-H_2, and 8-H_2), 1.52-1.66 (4H, m, 3-H_2, and 9-H_2), 1.86 (1H, m, 18-H), 2.09 (1H, m, 22-H), 2.05-2.22 (2H, m, 19-H_2), 2.18 (3H, s, 25-H_3), 2.24 (1H, dd, *J* 14.4, 8.1, 14-*H*H), 2.33 (2H, t, *J* 7.3, 2-H_2), 2.41 (2H, t, *J* 7.4, 10-H_2), 2.61 (1H, br d, *J* 14.4, 14-H*H*), 3.47 (1H, m, 16-H), 3.50-3.59 (2H, m, 23-H, and 26-*H*H), 3.71 (1H, m, 15-H), 3.81 (1H, m, 26-*H*H), 3.95 (1H, m, 17-H), 5.42 (1H, dd, *J* 15.4, 8.1, 21-H), 5.51 (1H, dd, *J* 15.4, 7.0, 20-H), 6.16 (1H, s, 12-H); δ_C(125 MHz, $CDCl_3$)

16.9 (C-27), 19.8 (C-25), 20.5 (C-24), 24.4 (C-9), 24.8 (C-3), 28.96 (CH_2), 29.04 (CH_2), 29.07 (CH_2), 29.12 (CH_2), 29.2 (CH_2), 32.5 (C-19), 33.8 (C-21), 42.2 (C-18), 43.4 (C-14), 44.5 (C-10), 44.9 (C-22), 65.1 (C-26), 69.1 (C-16), 70.5 (C-17), 71.5 (C-23), 75.1 (C-15), 125.2 (C-12), 129.8 (C-20), 134.6 (C-21), 155.2 (C-13), 177.6 (C-1), 202.0 (C-11); Found (ESI): 483.3318 $[M+H]^+$, (required $C_{27}H_{47}O_7$ 483.3316); Found (ESI): 505.3130 $[M+Na]^+$, (required $C_{27}H_{46}O_7Na$ 505.3136).

Reduction of carboxylic acid 11

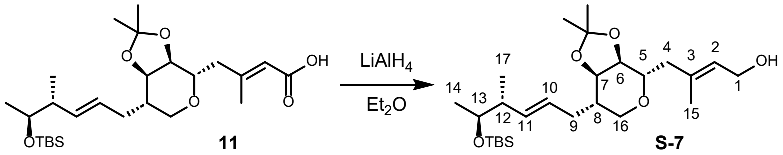

Lithium aluminium hydride (0.016 g, 0.417 mmol) was suspended in anhydrous Et_2O (0.76 mL) and cooled to 0 °C under an atmosphere of nitrogen. Acid **11** (0.096 g, 0.199 mmol) in anhydrous Et_2O (0.76 mL) was added dropwise, and then the reaction was stirred at room temperature for 16 h. After cooling to 0 °C, water (0.031 mL) was added and stirred for 5 min followed by the addition of $NaOH_{(aq)}$ (1 M, 0.062 mL). The mixture was stirred for 5 min, and then water (0.093 mL) was added. After stirring for another 5 min, the mixture was dried over $MgSO_4$, filtered and concentrated *in vacuo*. The crude product was purified by flash column chromatography (SiO_2, 10-20% EtOAc in petroleum ether 40-60 °C) giving alcohol **S-7** (0.074 g, 79%) as a colourless oil. $[\alpha]_D^{21}$ +7.0 (*c* 1.0, $CHCl_3$); ν_{max}/cm^{-1} 3430, 2957, 2928, 2897, 2858; δ_H(400 MHz, $CDCl_3$) 0.02 (6H, s, $Si(CH_3)_2$), 0.87 (9H, s, $SiC(CH_3)_3$), 0.95 (3H, d, *J* 6.9, 17-H_3), 1.02 (3H, d, *J* 6.2, 14-H_3), 1.34 and 1.49 (each 3H, each s, $C(CH_3)_2$), 1.71 (3H, s, 15-H_3), 1.96 (1H, m, 8-H), 2.08-2.26 (4H, m, 4-*H*H, 9-H_2, and 12-H), 2.37 (1H, dd, *J* 14.5, 2.2, 4-H*H*), 3.40 (1H, td, *J* 9.0, 3.4, 5-H), 3.60-3.72 (4H, m, 6-H, 16-H_2, and 13-H), 4.11 (1H, m, 7-H), 4.13 (1H, dd, *J* 12.2, 6.8, 1-*H*H), 4.18 (1H, dd, *J* 12.2, 7.3, 1-H*H*), 5.38 (1H, dt, *J* 15.4, 6.2, 10-H), 5.45 (1H, dd, *J* 15.4, 7.0, 11-H), 5.52 (1H, app. t, *J* 6.9, 2-H); δ_C(100 MHz, $CDCl_3$) –4.7 and –4.2 ($Si(CH_3)_2$), 16.1 (C-17), 16.8 (C-15), 18.3 (SiC), 20.7 (C-14), 26.0 ($SiC(CH_3)_3$), 26.5 and 28.5 ($C(CH_3)_2$), 34.2 (C-9), 37.1 (C-8), 43.1 (C-4), 44.4 (C-12), 59.4 (C-1), 66.8 (C-16), 71.9 (C-13), 74.5 (C-6), 75.5 (C-7), 76.6 (C-5), 108.6 ($C(CH_3)_2$), 125.8 (C-2), 127.4 (C-10), 135.6 (C-11), 136.7 (C-3); Found (ESI): 491.3166 $[M+Na]^+$, (required $C_{26}H_{48}O_5SiNa$ 491.3164).

Preparation of alkyne 19

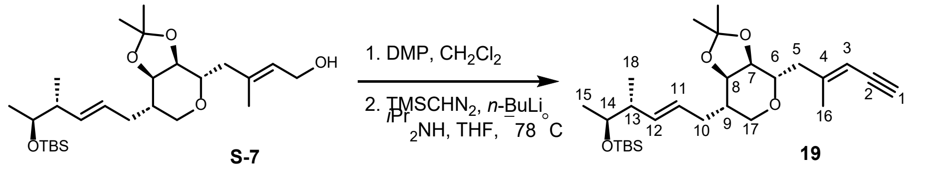

Alcohol **S-7** (0.055 g, 0.118 mmol) was dissolved in anhydrous CH_2Cl_2 (3 mL) under an atmosphere of nitrogen. Dess-Martin periodinane (15% wt, 0.366 mL) was added, and the reaction was stirred at room temperature for 1 h. Saturated $NaHCO_{3(aq)}$ (5 mL) was added, and the aqueous phase was extracted with Et_2O (3 × 20 mL). The combined organic layers were dried over $MgSO_4$, filtered and concentrated *in vacuo* to give crude aldehyde which was used to next step without further purification. Diisopropylamine (0.030 mL, 0.213 mmol) was dissolved in anhydrous THF (0.81 mL) and cooled to 0 °C under an atmosphere of nitrogen. *n*-BuLi (1.54 M, 0.115 mL, 0.177 mmol) was added dropwise, and the solution was stirred at 0 °C for 0.5 h followed by cooling to –78 °C. $TMSCHN_2$ (0.028 mL, 0.177 mmol) was added dropwise and then stirred at –78 °C for 0.5 h. A mixture of aldehyde from previous step in anhydrous THF (0.81 mL) was added dropwise to the reaction. The reaction was stirred at –78 °C for 1 h and then at room temperature for 2 h. Saturated $NH_4Cl_{(aq)}$ (5 mL) was added, and the aqueous phase was extracted with EtOAc (3 × 20 mL). The combined organic layers were dried over $MgSO_4$, filtered and concentrated *in vauco*. The crude product was purified by flash column chromatography (SiO_2, 10% EtOAc in petroleum ether 40-60 °C) giving alkyne **19** (0.044 g, 81% over 2 steps) as a colourless oil. $[\alpha]_D^{21}$ –0.10 (*c* 1.0, $CHCl_3$); ν_{max}/cm^{-1} 3314, 2957, 2928, 2857; δ_H(400 MHz, $CDCl_3$) 0.02 and 0.03 (each 3H, each s, $Si(CH_3)_2$), 0.88 (9H, s, $SiC(CH_3)_3$), 0.95 (3H, d, *J* 6.9, 18-H_3), 1.02 (3H, d, *J* 6.2, 15-H_3), 1.34 and 1.48 (each 3H, each s, $O_2C(CH_3)_2$), 1.97 (1H, m, 9-H), 1.95 (3H, s, 16-H_3), 2.10-2.24 (4H, m, 5-*H*H, 10-H_2, and 13-H), 2.44 (1H, d, *J* 14.7, 5-H*H*), 3.03 (1H, d, *J* 1.9, 1-H), 3.36 (1H, td, *J* 9.1, 2.7, 6-H), 3.61-3.71 (4H, m, 7-H, 14-H, and 17-H_2), 4.11 (1H, dd, *J* 4.8, 2.1, 8-H), 5.34-5.41 (2H, m, 3-H, and 11-H), 5.45 (1H, dd, *J* 15.5, 7.0, 12-H); δ_C(100 MHz, $CDCl_3$) –4.7 and –4.2 ($Si(CH_3)_2$), 16.1 (C-18), 18.3 (SiC), 20.0 (C-16), 20.7 (C-15), 26.0 ($SiC(CH_3)_3$), 26.5 and 28.5 ($O_2C(CH_3)_2$), 34.3 (C-10), 37.0 (C-9), 41.8 (C-4), 44.4 (C-13), 66.8 (C-17), 71.9 (C-14), 74.2 (C-7), 75.5 (C-8), 76.9 (C-6), 80.1 (C-1), 81.8 (C-2), 106.1 (C-3), 108.7 ($O_2C(CH_3)_2$), 127.3 (C-11), 135.7 (C-12), 151.2 (C-4); Found (ESI): 463.3231 $[M+H]^+$, (required $C_{27}H_{47}O_4Si$ 463.3238).

Preparation of 9-azidononanoic acid 20

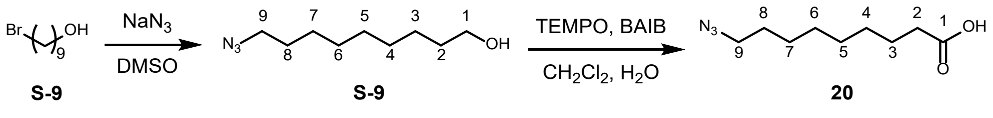

9-Bromononan-1-ol **S-8** (1.052 g, 4.713 mmol) was dissolved in anhydrous DMSO (10.4 mL) under an atmosphere of nitrogen. Sodium azide (0.337 g, 5.184 mmol) was added, and the reaction was stirred at room temperature for 16 h followed by the addition of water (20 mL). The aqueous phase was extracted with EtOAc (3 × 50 mL). The combined organic layers were dried over $MgSO_4$, filtered and concentrated *in vacuo*. The crude product was purified by flash column chromatography (SiO_2, 30% EtOAc in petroleum ether 40-60 °C) to give alcohol **S-9** (0.820 g, 94%) as a colourless oil. Rf = 0.49 (50% EtOAc in petroleum ether 40-60 °C); ν_{max}/cm^{-1} 3314, 3334, 2927, 2855, 2091; δ_H(400 MHz, $CDCl_3$) 1.27-1.41 (10 H, m, 3-H_2, 4-H_2, 5-H_2, 6-H_2, and 7-H_2), 1.51-1.64 (4H, m, 2-H_2, and 8-H_2), 3.25 (2H, t, *J* 7.0, 9-H_2), 3.63 (2H. t, *J* 6.6, 1-H_2); δ_C(100 MHz, $CDCl_3$) 25.8, 26.8, 29.0, 29.2, 29.4, 29.6 (C-3, C-4, C-5, C-6, C-7, C-8), 32.9 (C-2), 51.6 (C-9), 63.2 (C-1). Spectroscopic data in accord with the literature. [7]

Alcohol **S-9** (0.805 g, 4.345 mmol) was dissolved in CH_2Cl_2 (8.5 mL) and water (4 mL), and TEMPO (0.136 g, 0.869 mmol) and BAIB (3.498 g, 10.861 mmol) were added separately. The reaction was stirred vigorously for 16 h, and then water (20 mL) was added. The aqueous phase was extracted with EtOAc (3 × 50 mL). The combined organic layers were dried over $MgSO_4$, filtered and concentrated *in vacuo*. The crude product was purified by flash column chromatography (SiO_2, 20% EtOAc in petroleum ether 40-60 °C) giving acid **20** (0.859 g, 99%) as a colourless oil. ν_{max}/cm^{-1} 3039, 2930, 2857, 2092, 1705; δ_H(400 MHz, $CDCl_3$) 1.27- 1.42 (8H, m, 4-H_2, 5-H_2, 6-H_2, and 7-H_2), 1.54-1.68 (4H, m, 3-H_2, and 8-H_2), 2.35 (2H, t, *J* 7.5, 2-H_2), 3.25 (2H, t, *J* 7.0, 9-H_2); δ_C(100 MHz, $CDCl_3$) 24.7 (C-3), 26.8, 28.9, 29.05, 29.06, 29.2 (C-4, C-5, C-6, C-7, C-8), 34.1 (C-2), 51.6 (C-9). Spectroscopic data in accord with the literature. [8]

Preparation of acid S-10

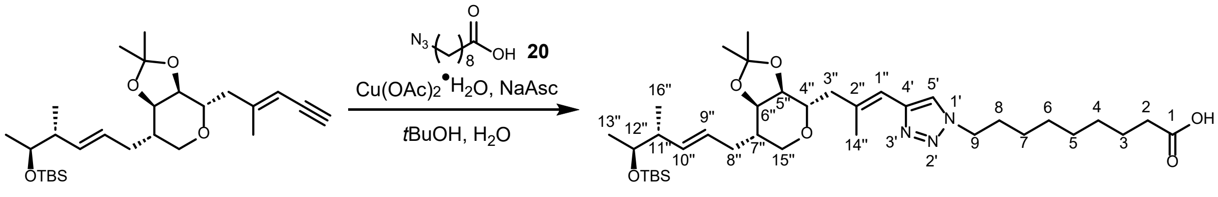

19 S-10

Alkyne **19** (0.042 g, 0.090 mmol) and azide **20** (0.019 g, 0.094 mmol) was dissolved in *t*BuOH (1.5 mL) and H_2O (1.5 mL). Sodium ascorbate (0.007, 0.036 mmol) and $Cu(OAc)_2$•H_2O (0.004, 0.018 mmol) were added, and then the mixture was heated to 80 °C. After stirring at 80 °C for 1 h, H_2O (5 mL) was added, and the aqueous phase was extracted with EtOAc (3 × 20 mL). The combined organic layers were dried over $MgSO_4$, filtered and concentrated *in vacuo*. The crude product was purified by flash column chromatography (SiO_2, 0-3% MeOH in CH_2Cl_2)

giving triazole **S-10** (0.054 g, 91%) as a colourless oil. $[\alpha]_D^{22}$ –0.10 (*c* 0.5, $CHCl_3$); ν_{max}/cm^{-1} 3139, 2954, 2928, 2856, 1723, 1711; δ_H(400 MHz, $CDCl_3$) 0.02 (6 H, s, $Si(CH_3)_2$), 0.87 (9H, s, $SiC(CH_3)_3$), 0.95 (3H, d, *J* 6.8, 16''-H_3), 1.02 (3H, d, *J* 6.1, 13''-H_3), 1.27-1.36 (8H, m, 4-H_2, 5-H_2, 6-H_2, and 7-H_2), 1.35 and 1.51 (each 3H, each s, $O_2C(CH_3)_2$), 1.56-1.66 (2H, m, 3-H_2), 1.84-1.94 (2H, m, 8-H_2), 1.97 (1H, m, 7''-H), 2.01 (3H, s, 14''-H_3), 2.10-2.36 (6H, m, 3''-*H*H, 8''-H_2, 11''-H, and 2-H_2), 2.56 (1H, br d, *J* 14.7, 3''-H*H*), 3.49 (1H, td, *J* 9.2, 2.5, 4''-H), 3.62-3.72 (3H, m, 12''-H, and 15''-H_2), 3.74 (1H, dd, *J* 8.7, 4.6, 5''-H), 4.13 (1H, dd, *J* 4.6, 1.7, 6''-H), 4.33 (2H, t, *J* 7.0, 9-H_2), 5.39 (1H, dt, *J* 15.5, 6.3, 9''-H), 5.45 (1H, dd, *J* 15.5, 7.2, 10''-H), 6.39 (1H, s, 1''-H), 7.41 (1H, s, 5'-H); δ_C(100 MHz, $CDCl_3$) –4.7 and –4.2 ($Si(CH_3)_2$), 16.0 (C-16''), 18.3 (SiC), 19.4 (C-14''), 20.7 (C-13''), 24.7 (C-3), 26.0 ($SiC(CH_3)_3$), 26.4, 26.5, 28.6, 28.8, 28.9, 29.0 ($O_2C(CH_3)_2$, C-4, C-5, C-6, C-7), 30.4 (C-8), 34.3 (C-8'', C-2), 37.1 (C-9), 43.8 (C-3''), 44.4 (C-11''), 50.3 (C-9), 66.8 (C-15''), 71.9 (C-12''), 74.4 (C-5''), 75.5 (C-6''), 77.0 (C-4''), 108.7 ($O_2C(CH_3)_2$), 116.1 (C-1''), 121.2 (C-5'), 127.4 (C-9''), 135.6 (C-10''), 137.8 (C-2''), 145.7 (C-4'), 178.3 (C-1); Found (ESI): 662.4571 $[M+H]^+$, (required $C_{36}H_{64}N_3O_6Si$ 662.4559); Found (ESI): 684.4387 $[M+Na]^+$, (required $C_{36}H_{63}N_3O_6SiNa$ 684.4378).

Deprotection of S-10 to give 21

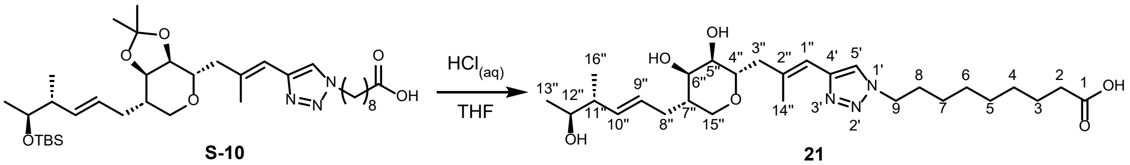

Acid **S-10** (0.060 g, 0.091 mmol) was dissolved in THF (0.7 mL) and H_2O (0.7 mL), and then $HCl_{(aq)}$ (2 M, 0.38 mL) was added dropwise. The solution was stirred for 16 h and then diluted by H_2O (10 mL) and EtOAc (20 mL). The aqueous phase was extracted with EtOAc (5 × 20 mL). The combined organic layers were dried over $MgSO_4$, filtered and concentrated *in vacuo*. The crude product was purified by flash column chromatography (SiO_2, 10% MeOH in CH_2Cl_2) giving acid **21** (0.035 g, 76%) as a colourless oil. $[\alpha]_D^{22}$ –15.0 (*c* 1.0, MeOH); ν_{max}/cm^{-1} 3373, 2924, 2855, 1714, 1666; δ_H(500 MHz, CD_3OD) 0.99 (3H, d, *J* 6.9, 16''-H_3), 1.09 (3H, d, *J* 6.4, 13''-H_3), 1.29-1.39 (8H, m, 4-H_2, 5-H_2, 6-H_2, 7-H_2), 1.54-1.62 (2H, m, 3-H_2), 1.78 (1H, m, 7''-H), 1.88-1.95 (2H, m, 8-H_2), 1.98 (3H, s, 14''-H_3),2.11-2.22 (3H, m, 8''-H_2, 11''-H), 2.23-2.32 (3H, m, 3''-*H*H, and 2-H_2), 2.70 (1H, d, *J* 14.2, 3''-H*H*), 3.41 (1H, dd, *J* 8.9, 2.8, 5''-H), 3.51 (1H, d, *J* 11.4, 15''-*H*H), 3.61 (1H, qd, *J* 6.2, 5.2, 12''-H), 3.76 (1H, dd, *J* 9.2, 2.1, 4''-H), 3.80 (1H, dd, *J* 11.4, 2.6, 15''-H*H*), 3.85 (1H, m, 6''-H), 4.39 (2H, t, *J* 7.1, 9-H_2), 5.40-5.49 (2H, m, 9''-H, and 10''-H), 6.33 (1H, s, 1''-H), 7.93 (1H, s, 5'-H); δ_C(125 MHz, CD_3OD) 16.6 (C-16''), 19.3 (C-14''), 20.3 (C-13''), 26.1 (C-3), 27.4, 29.9, 30.1, 30.2 (C-4, C-5, C-6, C-7), 31.3 (C-8), 33.7 (C-8''), 44.57, 44.59 (C-3'', C-7''), 45.3 (C-11''), 51.3 (C-9), 65.6 (C-15''), 70.0 (C-5''), 71.6

(C-6”), 72.1 (C-12”), 76.6 (C-4”), 116.6 (C-1”), 123.3 (C-5’), 129.7 and 135.7 (C-9”, C-10”), 140.0 (C-2”), 146.6 (C-4’), 178.1 (C-1); Found (ESI): 508.3367 $[M+H]^+$, (required $C_{27}H_{46}N_3O_6$ 508.3381); Found (ESI): 530.3187 $[M+Na]^+$, (required $C_{27}H_{45}N_3O_6Na$ 530.3201).

Synthesis of 22

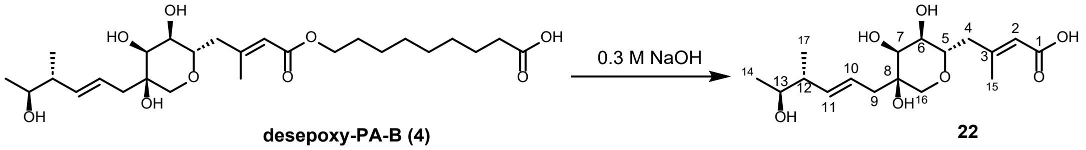

Crude desepoxyPA-B **4** (0.486 g, 0.98 mmol) was dissolved in 0.3 M NaOH (125 mL) and stirred for 24 hours at room temperature. The reaction mixture was concentrated to ~50 mL, acidified to ~pH 2 with 2 M HCl. The resulting solution was extracted with EtOAc (3 x 150 mL) then the combined organic layers were dried over $MgSO_4$, and the solvent was removed *in vacuo*. The crude material was purified by flash column chromatography (98% EtOAc/pet.ether, with 1 mL AcOH per 100 mL of eluent) to afford acid **22** (0.286 g, 59%) as an orange oil; δ_H(400 MHz, CD_3OD) 5.71 (1H, br s, 2-H), 5.56 (1H, dt, *J* 15.4, 7.0, 10-H), 5.45 (1H, dd, *J* 15.5, 7.6, 11-H), 3.70 (1H, d, *J* 3.0, 7-H), 3.65 – 3.55 (2H, m, 5-H and 13-H), 3.42 (1H, d, *J* 10.9, 16-*H*H), 3.33 (1 H, m, 6H*H*), 3.31 (1H, d, *J* 1.4, 6-H), 2.64 (1H, d, *J* 14.5, 4-*H*H), 2.34 – 2.27 (2H, m, 9-*H*H and 12-H), 2.18 (1H d, *J* 6.1, 9-H*H*), 2.17 – 2.11 (4H, m, 4-H*H* and 15-H_3), 1.09 (3H, d, *J* 6.4, 14-H_3), 0.99 (3H, d, *J* 6.9, 17-H_3); δ_C(100 MHz, CD_3OD) 170.1(C-1), 158.9 (C-3), 137.3 (C-11), 125.8 (C-10), 118.5 (C-2), 75.1 (C-7), 74.0 (C-6), 72.7 (C-8), 72.1 (C-5), 70.8(C-16), 69.6 (C-13), 45.4 (C-12), 43.9 (C-4), 39.6 (C-9), 20.2 (C-15), 19.2 (C-14), 16.4 (C-17). NMR data consistent with literature. [4]

Protection of tetrol 22

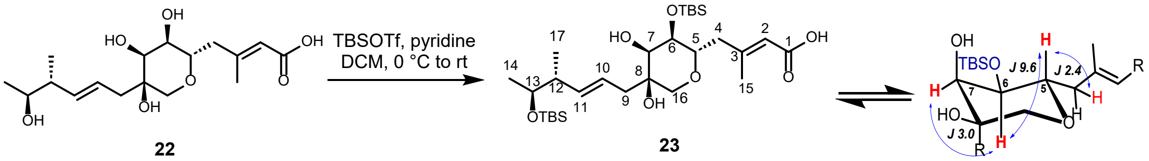

Monic acid **22** (0.450 g, 1.31 mmol) was dissolved in DCM (12.25 mL) and pyridine (2.45 mL, 30.36 mmol) and cooled to 0 °C, then TBSOTf (2.12 mL, 9.82 mmol) was added dropwise over 5 minutes. The reaction mixture was stirred at room temperature for 1 h then quenched with water (16 mL) and acidified with 2 M HCl (4 mL). The layers were separated and the aqueous extracted with DCM (3 x 20 mL). The combined organic layers were dried over $MgSO_4$ and the solvent removed *in vacuo*. The crude material was dissolved in THF (1.63 mL) and MeOH (0.81 mL) then K_2CO_3 (0.361 g, 2.61 mmol) in water (0.81 mL) was added dropwise. The reaction mixture was stirred at room temperature for 20 hours then quenched

with water (8 mL) and 1 M HCl (8 mL). The resulting solution was extracted with EtOAc (3 x 40 mL) and the combined organic layers were dried over Na_2SO_4 and the solvent removed *in vacuo*. The crude material was purified by flash column chromatography (30% EtOAc in petroleum ether 40-60 °C + 0.05% AcOH) to afford silyl protected monic acid **23** (0.130 g, 30%) as a colourless oil; $[\alpha]_D^{20}$ = -14.0 (*c* 1, $CHCl_3$); ν_{max} (film) 3435, 2956, 2929, 2858, 1693, 1643, 836, 754; δ_H(400 MHz, $CDCl_3$) 5.76 (1H, s, 2-H), 5.61 – 5.41 (2H, m, 10-H, and 11-H), 3.75 (1H, d, *J* 3.0, 7-H), 3.70 (1H, qd, *J* 6.1, 4.0, 13-H), 3.61 (1H, td, *J* 9.6, 2.4, 5-H), 3.48 (1H, m, 6-H), 3.45 - 3.40 (2H, m, 16-H_2), 2.52 (1H, d, *J* 14.0, 4-*H*H), 2.36 (2H, m, 9-*H*H and 12-H), 2.34 (1H, m, 9-H*H*), 2.18 (3H, br s, 15-H_3), 2.10 (1H, m, 7-OH), 1.99 (1H, d, *J* 2.4, 4-H*H*), 1.03 (3H, d, *J* 6.2, 14-H_3), 0.98 (3H, d, *J* 6.9, 17-H_3), 0.90 (9H, s, $SiC(CH_3)_3$), 0.88 (9H, s, $SiC(CH_3)_3$), 0.12 (6H, s, $Si(CH_3)_2$), 0.03 (3H, s, $SiCH_3$), 0.02 (3H, s, $SiCH_3$); δ_C(101 MHz, $CDCl_3$) 171.7 (C-1), 159.8 (C-3), 137.0 (C-11), 124.2 (C-10), 117.4 (C-2), 74.1 (C-7), 72.6 (C-5), 72.0 (C-8), 71.7 (C-6), 71.5 (C-16), 70.0 (C-13), 44.7 (C-12), 43.4 (C-4), 38.4 (C-9), 26.1 ($SiC(CH_3)_3$), 25.9 ($SiC(CH_3)_3$), 20.8 (C-14), 19.4 (C-15), 18.3 ($SiC(CH_3)_3$), 18.1 ($SiC(CH_3)_3$), 16.6 (C-17), -3.9 ($SiCH_3$), -4.2 ($SiCH_3$), -4.5 ($SiCH_3$), -4.6 ($SiCH_3$); HRMS (ESI) calc. for $[C_{29}H_{57}O_7Si_2]^+$ 573.3637 Found 573.3640.

Esterification of acid 23 to give ester 25

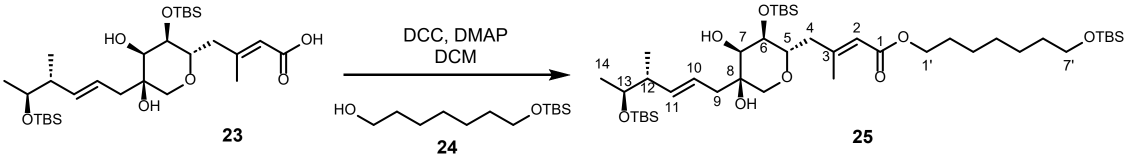

23 24 25

Carboxylic acid **23** (0.050 g, 0.09 mmol) was dissolved in DCM (1 mL) under nitrogen then DCC (39 mg, 0.19 mmol), DMAP (3 mg, 0.02 mmol), and alcohol **24** (0.052 g, 0.21 mmol) in DCM (1 mL) were added sequentially. The reaction mixture was stirred at room temperature for 24 hours, filtered, and the solvent removed *in vacuo*. The crude material was purified by flash column chromatography (10% EtOAc in petroleum ether) to give protected ester **25** (0.042 g, 60%) as a colourless oil; $[\alpha]_D^{20}$ = -16.0 (*c* 1, $CHCl_3$); ν_{max} (film) 3511, 2961, 2929, 2857, 2119, 1717, 1649, 1255, 1098, 835; δ_H (400 MHz, $CDCl_3$) 5.72 (1H, s, 2-H), 5.54 (1H, m, 10-H), 5.45 (1H, dd, *J* 14.9, 8.5, 11-H), 4.06 (2H, t, *J* 6.7, 1'-H_2), 3.73 (1H, m, 7-H), 3.69 (1H, m, 13-H), 3.63 – 3.56 (3H, m, 5-H and 7'-H_2), 3.49 – 3.43 (2H, m, 6-H and 16-*H*H), 3.39 (1H, d, *J* 11.5, 16-H*H*), 2.76 (1H, s, OH), 2.56 (1H, s, OH), 2.49 (1H, d, *J* 14.5, 4-*H*H), 2.34 (2H, d, *J* 6.8, 9-H_2), 2.22 – 2.10 (5H, m, 15-H_3, 4-H*H* and 12-H), 1.68 – 1.57 (2H, m, 2'-H_2), 1.55 – 1.45 (2H, m, 6'-H_2), 1.38 – 1.29 (6H, m, 3'-H_2, 4'-H_2 and 5'-H_2), 1.03 (3H, d, *J* 6.2, 14-H_3), 0.97 (3H, d, *J* 6.9, 17-H_3), 0.91 (9H, s, $SiC(CH_3)_3$), 0.88 (9H, s, $SiC(CH_3)_3$), 0.80 (9H, s, $SiC(CH_3)_3$), 0.10 (6H, s, $Si(CH_3)_2$), 0.04 (6H, s, $Si(CH_3)_2$), 0.03 (3H, s, $SiCH_3$), 0.02 (3H, s, $SiCH_3$); δ_C (101 MHz, $CDCl_3$) 166.9 (C-1), 156.6 (C-3), 137.0 (C-11), 124.3 (C-10), 117.9 (C-2), 74.0 (C-7), 72.5 (C-5), 72.0 (C-8), 71.7 (C-6), 71.6 (C-16), 70.0 (C-13), 64.0 (C-1'), 63.4 (C-7'), 44.7 (C-12), 43.2

(C-4), 38.4 (C-9), 33.0 (C-6'), 29.3 (C-4'), 28.9 (C-2'), 26.2 (C-3'), 26.1 (SiC(CH_3)$_3$), 26.0 (SiC(CH_3)$_3$), 25.9 (C-5'), 25.8 (SiC(CH_3)$_3$), 20.8 (C-14), 19.0 (C-15), 18.6 (Si*C*(CH_3)$_3$), 18.3 (Si*C*(CH_3)$_3$), 18.1 (Si*C*(CH_3)$_3$), 16.6 (C-17), -3.9 ($SiCH_3$), -4.2 ($SiCH_3$), -4.5 ($SiCH_3$), -4.6 ($SiCH_3$), -5.1 ($Si(CH_3)_2$); HRMS (ESI) calc. for $[C_{42}H_{85}O_8Si_3]^+$ 801.5547 Found 801.5557.

Deprotection of silyl ether 25 to give 26

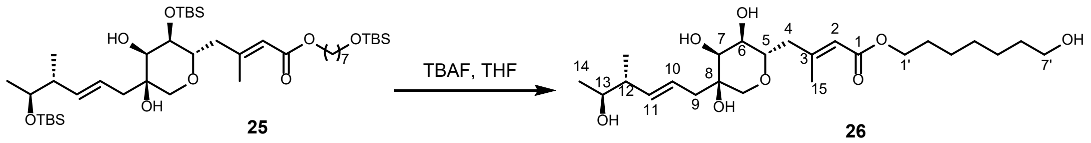

Protected monate ester **25** (0.112 g, 0.14 mmol) was cooled to 0 °C under nitrogen then TBAF (1 M in THF, 3.50 mL, 3.50 mmol) was added dropwise. The reaction mixture was stirred at room temperature for 24 hours then diluted with water (15 mL), brine (5 mL) and EtOAc (30 mL). The organic layer was separated and the aqueous extracted with further EtOAc (3 x 30 mL). The combined organic layers were dried over Na_2SO_4 and the solvent removed *in vacuo*. The crude material was purified by flash column chromatography (EtOAc) to give alcohol **26** (0.050 g, 47%) as a colourless oil; $[\alpha]_D^{21}$ = +20.0 (*c* 0.5, $CHCl_3$); ν_{max} (film) 3388, 2931, 2859, 1698, 1646, 905, 726; δ_H (600 MHz, $CDCl_3$) 5.75 (1H, br. s, 2-H), 5.64 – 5.37 (2H, m, 10-H and 11-H), 4.08 (2H, t, *J* 6.6, 1'-H_2), 3.81 (1H, d, *J* 3.1, 7-H), 3.62 (3H, m, 13-H and 7'-H_2), 3.52 (1H, m, 5-H), 3.46 (1H, d, *J* 11.2, 16-*H*H), 3.41 – 3.37 (2H, m, 6-H and 16-H*H*), 2.63 (1H, d, *J* 14.8, 4-*H*H), 2.34 (1H, m, 9-H_2), 2.19 (3H, s, 15-H_3), 2.09 – 2.00 (2H, m, 12-H, and 4-H*H*), 1.70 – 1.60 (2H, m, 2'-H_2), 1.61 – 1.47 (2H, m, 6'-H_2), 1.40 – 1.29 (6H, m, 3'-H_2, 4'-H_2 and 5'-H_2), 1.18 (3H, d, *J* 6.3, 14-H_3), 1.00 (3H, d, *J* 6.8, 17-H_3); δ_C (151 MHz, $CDCl_3$) 169.0 (C-1), 156.8 (C-3), 138.0 (C-11), 125.1 (C-10), 117.8 (C-2), 74.6 (C-7), 72.5 (C-5), 71.8 (C-8), 71.4 (C-6), 70.1 (C-16), 69.2 (C-13), 64.0 (C-1'), 63.1 (C-7'), 45.2 (C-12), 42.9 (C-4), 38.5 (C-9), 32.6 (C-6'), 29.1 (C-4'), 28.7 (C-2'), 26.1 (C-3'), 25.7 (C-5'), 20.9 (C-14), 19.4 (C-15), 17.0 (C-17); HRMS (ESI) calc. for $[C_{24}H_{43}O_8]^+$ 459.2942 Found 459.2944.

General procedure of whole-cell biotransformation with MupW strain

Whole-cell biotransformation with MupW gene was amplified from the plasmid pOPINF (prelinearized with KpnI and HindIII) using the In-Fusion HD Cloning Kit (Clonetech).[9] The resulting plasmids were verified by sequencing and transformed into *E. coli* BL21 (DE3) for expression. Protein expression was carried out with auto induction medium LB broth base including trace elements (FORMEDIUM™). For *in vivo* biotransformations, 50 mL of overnight

culture of *E. coli* BL21 (DE3) cells overexpressing MupW was centrifuged at 6000 rpm for 5 min, cell pellets were resuspended in 2 mL of 100 mM potassium phosphate buffer pH 7.2 supplemented with 20 mM glucose, substrates dissolved in MeOH were then added and the reactions were incubated at 30 °C, 180 rpm for 48 h. Reactions were quenched by adding equal volume of acetonitrile, vortexed and centrifuged. The acetonitrile layer was injected for LC-MS analysis and products were purified by preparative HPLC for NMR analysis.

Oxidation of C_7-desepoxy-PA-B (27)

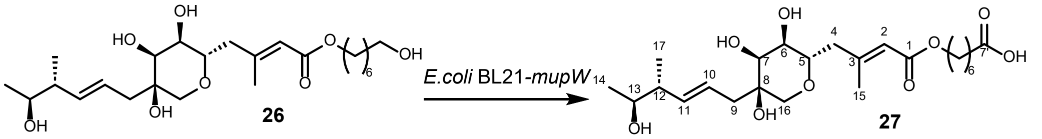

Whole cell biotransformation of primary alcohol **26** (0.008 g) was conducted utilising the same protocol as described in general procedure to give acid **27;** (0.0036 g, 45%); $[\alpha]_D^{21}$ = 5.0 (*c* 0.4, $CHCl_3$); v_{max} (film) 3404, 2957, 2925, 2858, 2229,1713, 1652; δ_H (600 MHz, CD_3OD) 5.75 (1H, s, 2-H), 5.58 (1H, dt, *J* 15.5, 7.2, 10-H), 5.48 (1H, dd, *J* 15.5, 7.9, 11-H), 4.08 (2H, t, *J* 6.6, 1'-H_2), 3.73 (1H, d, *J* 2.9, 7-H), 3.67 – 3.59 (2H, m, 13-H and 5-H), 3.44 (1H, d, *J* 10.9, 16-*H*H), 3.37 – 3.33 (2H, m, 6-H and 16-H*H*), 2.66 (1H, d, *J* 14.5, 4-*H*H), 2.33 (2H, d, *J* 7.2, 9-H_2), 2.27 (2H, t, *J* 7.4, 6'-H_2), 2.21 – 2.16 (5H, m, 12-H, 4-H*H* and 15-H_3), 1.69 – 1.59 (4H, m, 2'-H_2 and 5'-H_2), 1.43 – 1.36 (4H, m, 3'-H_2 and 4'-H_2), 1.11 (3H, d, *J* 6.3, 14-H_3), 1.01 (3H, d, *J* 6.8, 17-H_3); δ_C (151 MHz, CD_3OD) 178.1 (C-7'), 168.0 (C-1), 158.6 (C-3), 137.0 (C-11), 125.4 (C-10), 117.8 (C-2), 74.8 (C-5), 73.6 (C-7), 72.3 (C-8), 71.7 (C-13), 70.4 (C-6), 69.2 (C-16), 64.3 (C-1'), 45.0 (C-12), 43.5 (C-4), 39.2 (C-9), 35.1 (C-6'), 29.5 (C-3' or C-4'), 29.2 (C-2'), 26.4 (C-3' or C-4'), 25.8 (C-5'), 19.8 (C-14), 18.9 (C-15), 16.0 (C-17); HRMS (ESI) calc. for $[C_{24}H_{40}O_9]^+$ 473.2745 Found 473.2725.

General experimental procedures for feeding substrates to cultures of *P. fluorescens*

P. fluorescens mutant strains were incubated on an L-agar plate at 30 ºC for 24 hours. Using a toothpick, 25 ml of a modified L-medium in a 100 ml flask was inoculated with as much bacteria as possible and grown at 22 ºC, 200 rpm for 60 hours. The compounds isolated from mutant strains were added to the flasks immediately after inoculation. The cells were separated from the medium by centrifugation (7000 rpm for 10 mins). The medium was extracted with EtOAc (0.5 v/v) 2 times. The crude extract obtained from the medium was filtered with cotton and then analysed by LCMS and HPLC (HPLC column: Phenomenex Luna column, 250 x 3.0 mm, 5µm, 40 ºC, and flow: 0.7 mL/min). [1, 2]

Feeding desepoxy PA-B (4) and 27 to *ΔmupW* or *ΔmupH* of *P. fluorescens* mutant

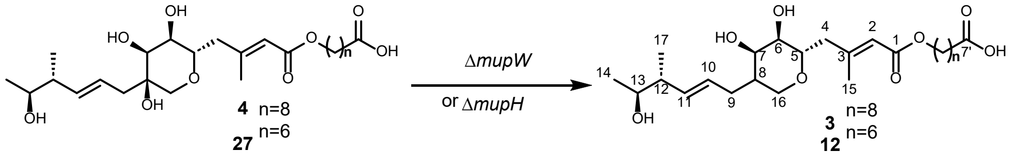

A 50 mL scale fermentation of *ΔmupW* or *ΔmupH P. fluorescens* was carried out as the per general procedure. 0.0016 g of **4** or **27** dissolved in 50 µL of MeOH and fed to a *ΔmupW* mutant immediately after inoculation. A total of 0.004 g of the substrate (**4** or **27**) was added to the culture in four portions over two days of fermentation. After incubation the culture was extracted with EtOAc to give a crude extract, which was subjected to LC-MS analysis. PA-C **3** was consistent with previous data, [1] product **12** was purified by preparative reverse phase HPLC employing a 15 – 60% isocratic water/MeCN method with 0.05% formic acid solvent system over 20 minutes; $[\alpha]_D^{21}$ = 4.0 (*c* 0.5, $CHCl_3$); ν_{max} (film) 3388, 2930, 2925, 2838, 2219, 1716; δ_H(600 MHz, CD_3OD) ; δ_H(600 MHz, CD_3OD) 5.77 (1H, s, 2-H), 5.48 – 5.45 (2H, m, 10-H and 11-H), 4.10 (2H, t, *J* 6.6, 1'-H_2), 3.86 (1H, t, *J* 3.3, 7-H), 3.80 (1H, dd, *J* 11.5, 2.9, 16-*H*H), 3.75 (1H, td, *J* 9.4, 2.6, 5-H), 3.68 – 3.59 (2H, m, 13-H, 16-H*H*), 3.53 (1H, d, *J* 11.5, 6-H), 2.68 (1H, d, *J* 14.4, 4-*H*H), 2.29- 2.23 (3H, m, 6'-H_2 and 4-H*H*), 2.20 (3H, s, 15-H_3), 2.19 – 2.14 (3H, m, 9-H_2, and 12-H), 1.79 (1H, m, 8-H), 1.72 – 1.60 (4H, m, 2'H_2 and 5'H_2), 1.45 – 1.39 (4H, m, 3'-H_2 and 4'-H_2), 1.12 (3H, d, *J* 6.4, 14-H_3), 1.01 (3H, d, *J* 6.9, 17-H_3); δ_C(151 MHz, CD_3OD) 178.1 (C-7'), 167.0 (C-1), 157.6 (C-3), 135.3 (C-11), 129.2 (C-10), 117.8 (C-2), 75.6 (C-5), 71.6 (C-13), 71.1 (C-7), 69.4 (C-6), 65.2 (C-16), 64.3 (C-1') 44.8 (C-12), 43.6 (C-4), 43.2 (C-8), 33.2 (C-6'), 30.2 (C-9), 29.5 (C-3', or C-4'), 29.2 (C-2'), 26.6 (C-3', or C-4'), 26.4 (C-5'), 19.8 (C-14), 18.8 (C-15), 16.2 (C-17); HRMS (ESI) calc. for $[C_{24}H_{40}O_8]^+$ 457.2756 Found 457.2735.

4. NMR Spectra

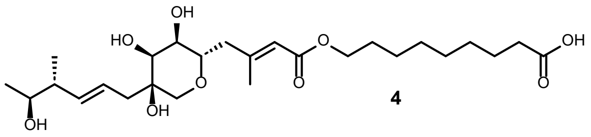

4

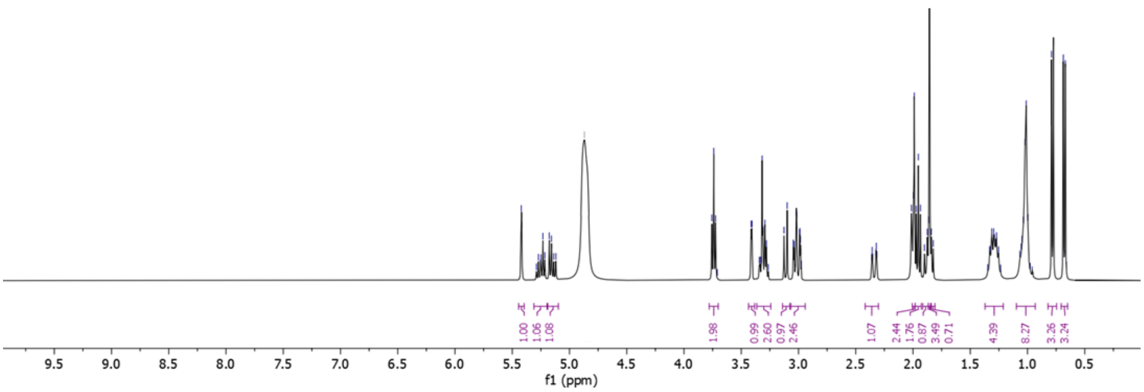

Fig.1: Desepoxy-PA-B (**4**) ^{1}H-NMR (400 MHz, CD_3OD)

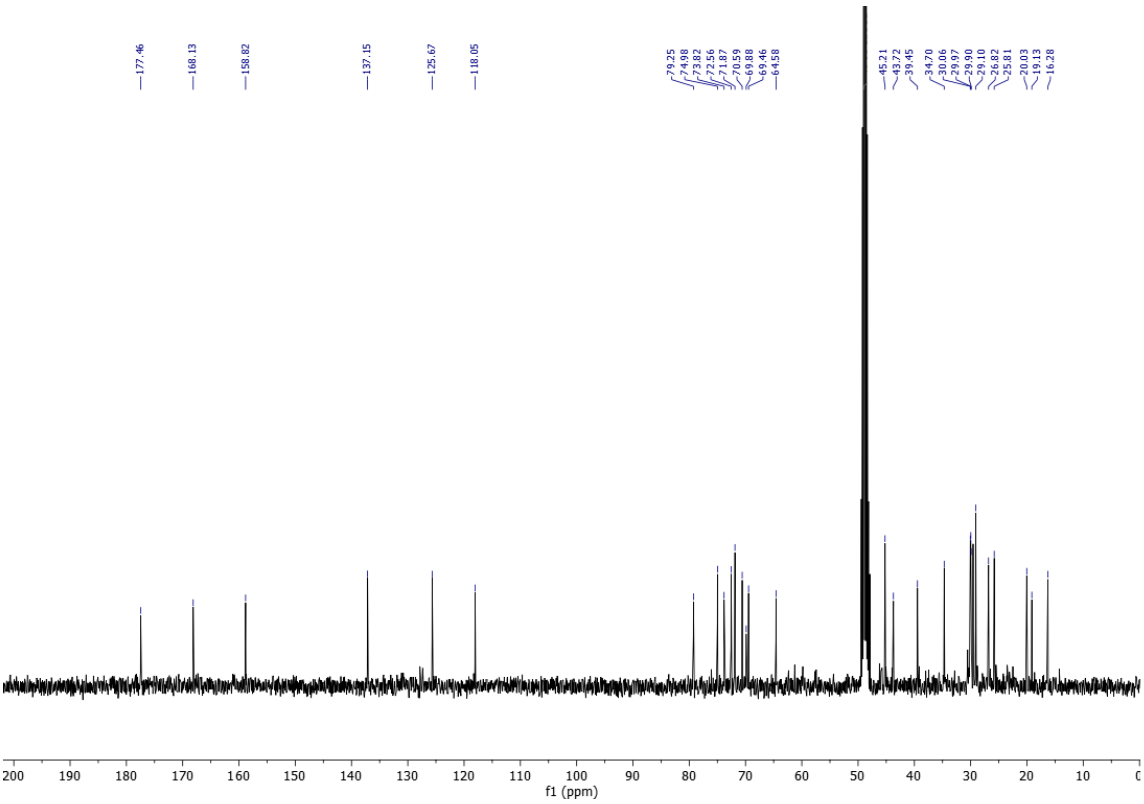

Fig.2: DesepoxyPA-B (**4**) ^{13}C-NMR (100 MHz, CD_3OD)

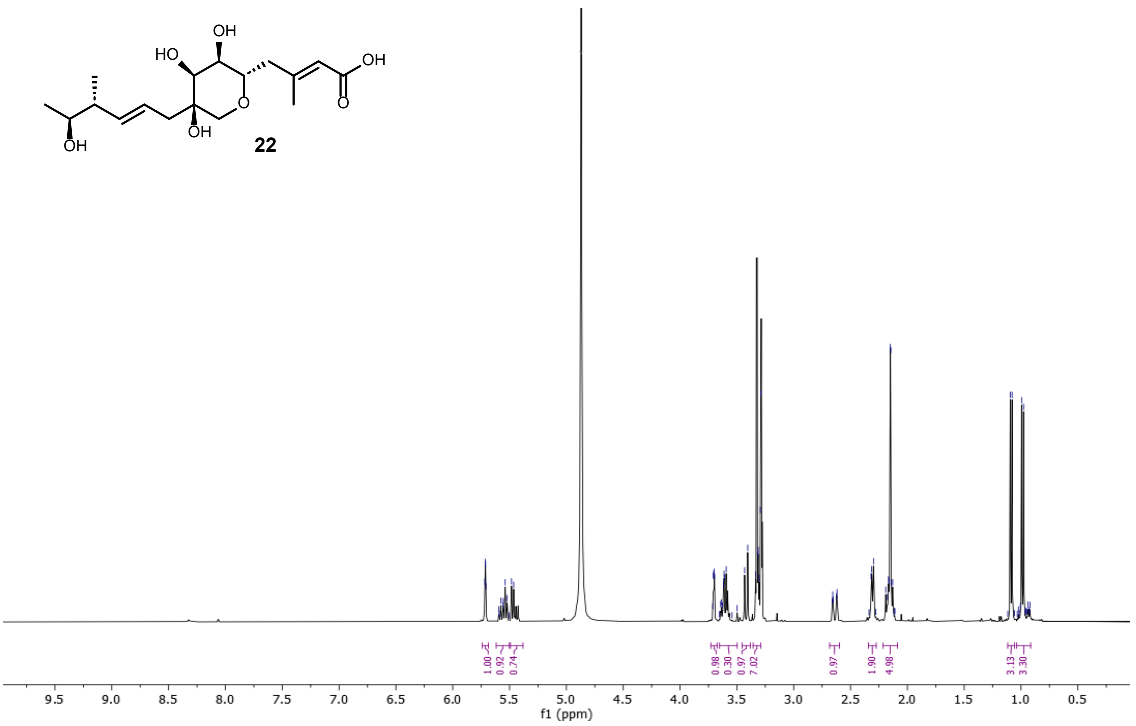

Fig.3: Truncated acid **22** ^{1}H-NMR (400 MHz, CD_3OD)

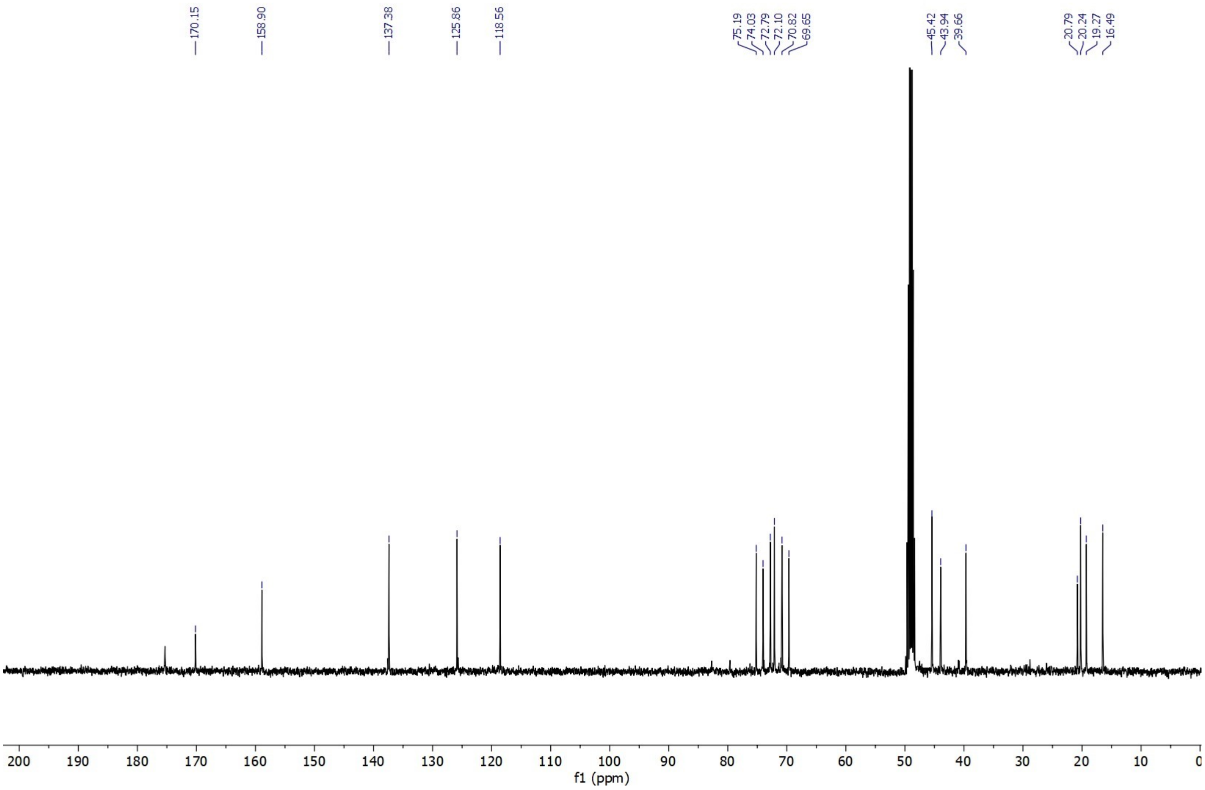

Fig.4: Truncated acid **22** ^{13}C-NMR (100 MHz, CD_3OD)

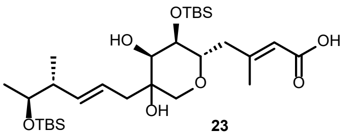

23

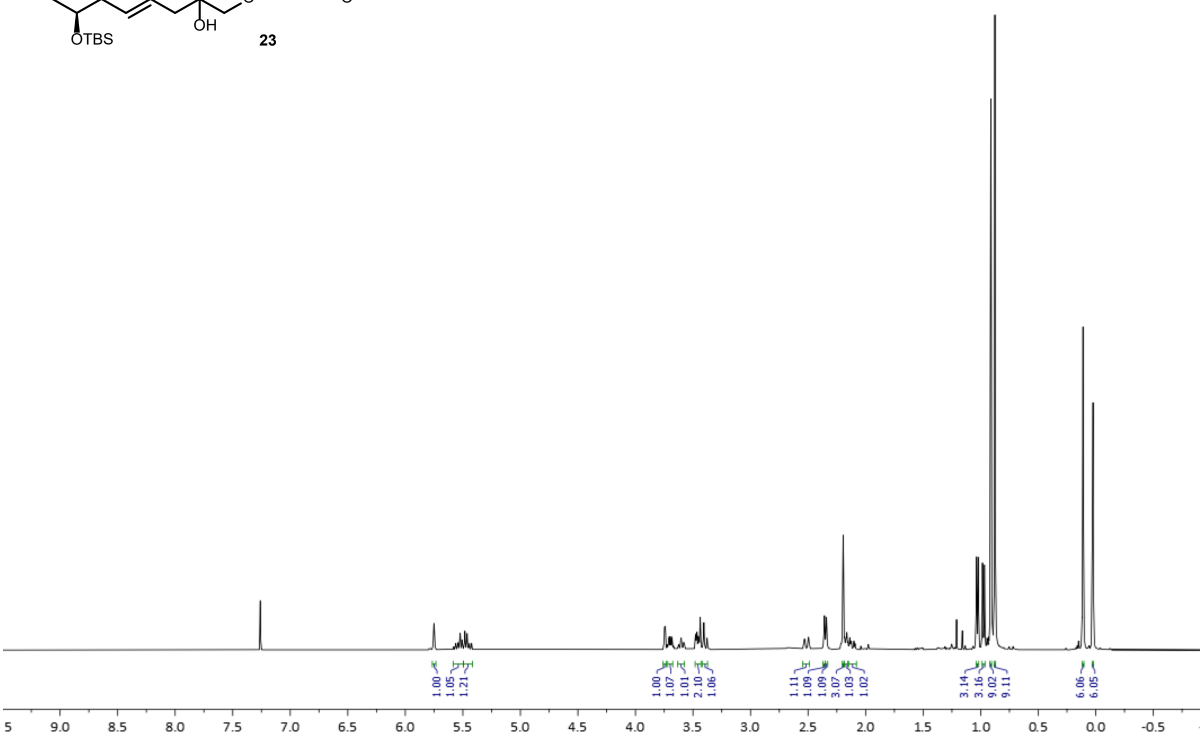

Fig.5: Silyl protected product **23** ^{1}H-NMR (400 MHz, $CDCl_3$)

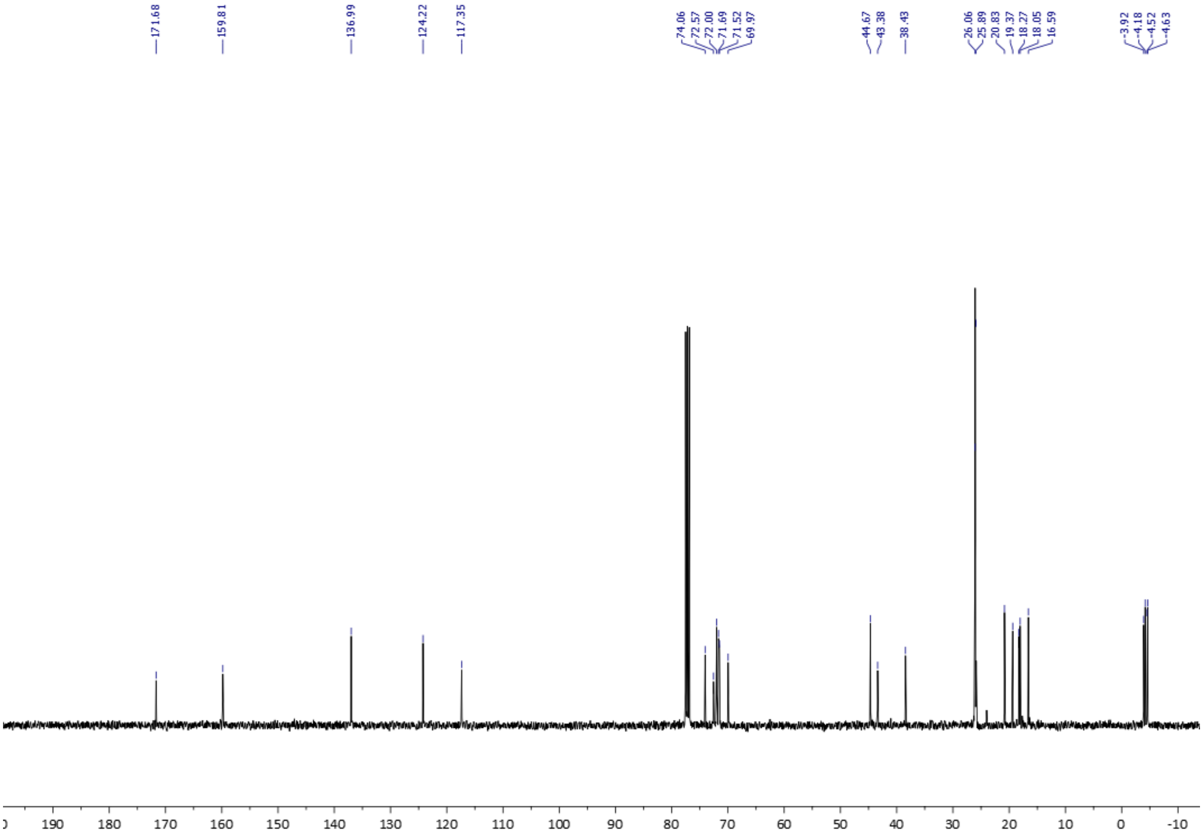

Fig.6: Silyl protected product **23** ^{13}C-NMR (101 MHz, $CDCl_3$)

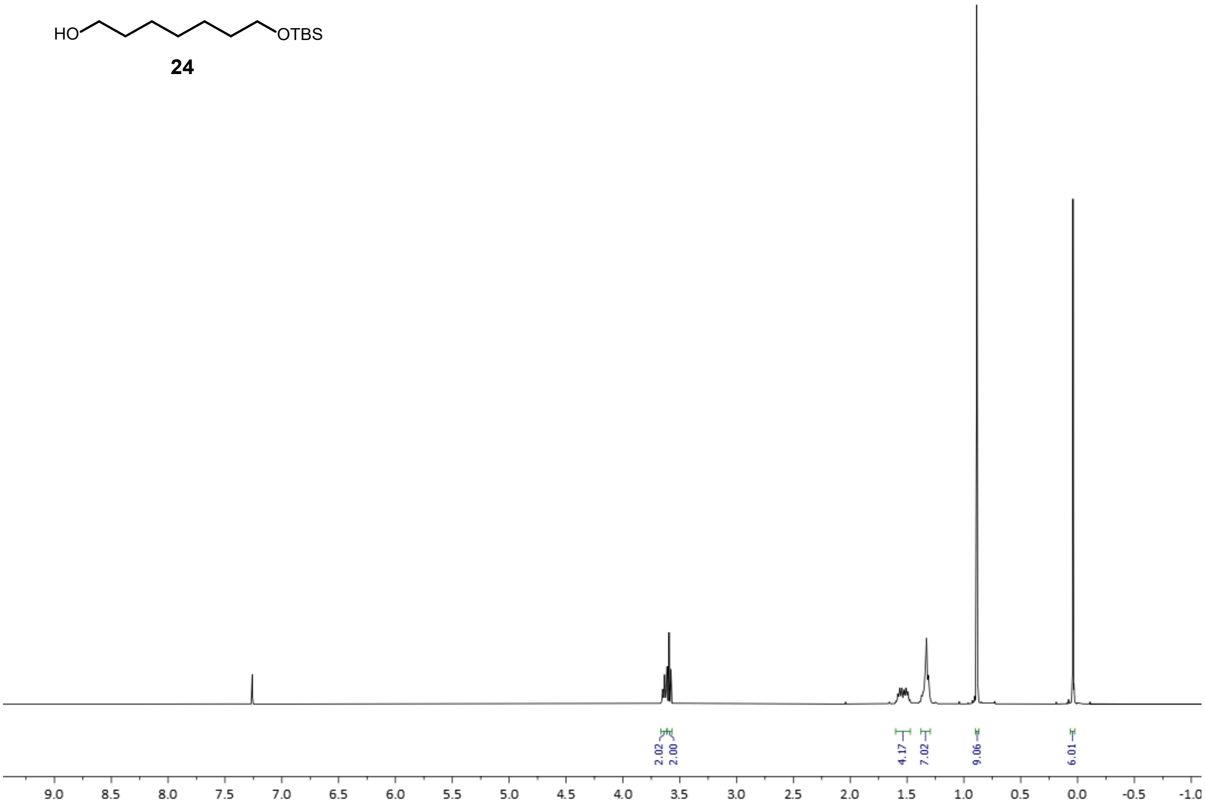

Fig.7: Silyl ether **24**) ^{1}H-NMR (400 MHz, $CDCl_3$)

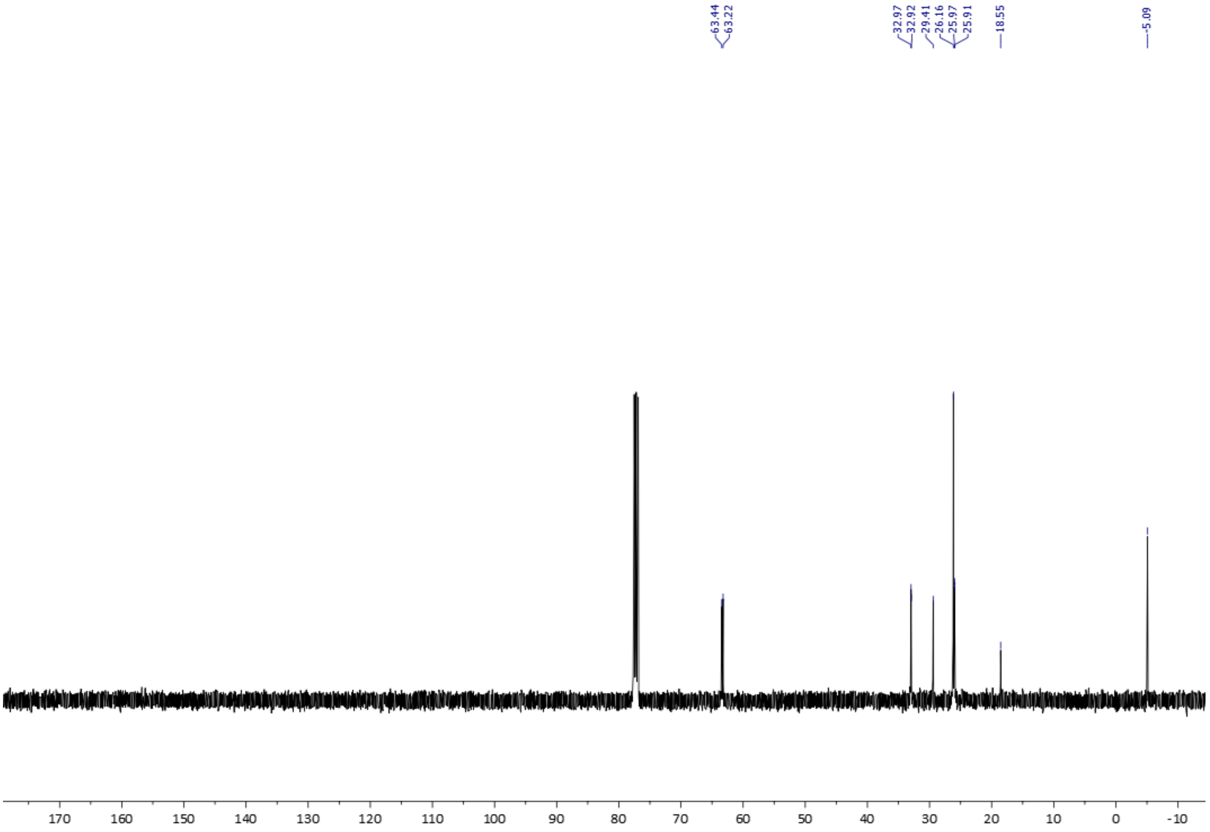

Fig.8: Silyl ether **24** ^{13}C-NMR (101 MHz, $CDCl_3$)

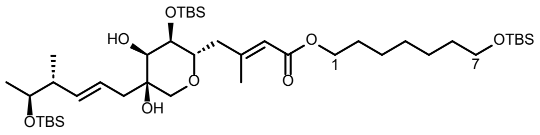

25

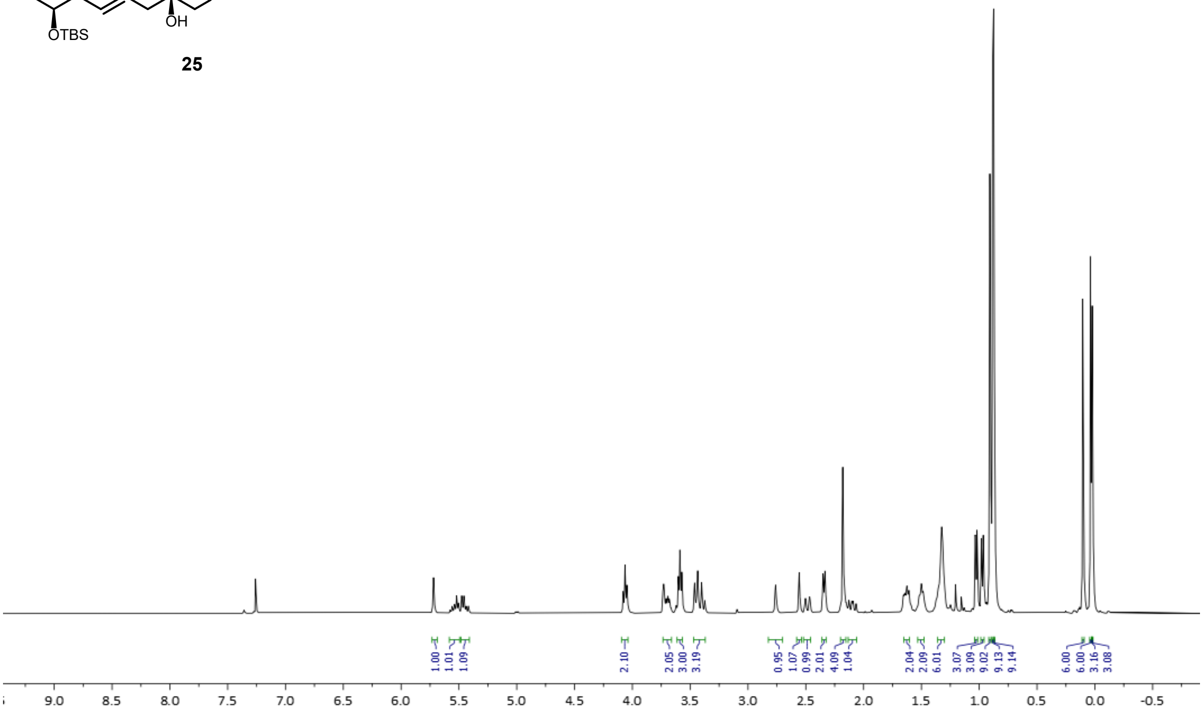

Fig.9: Ester (**25**) ^{1}H-NMR (400 MHz, $CDCl_3$)

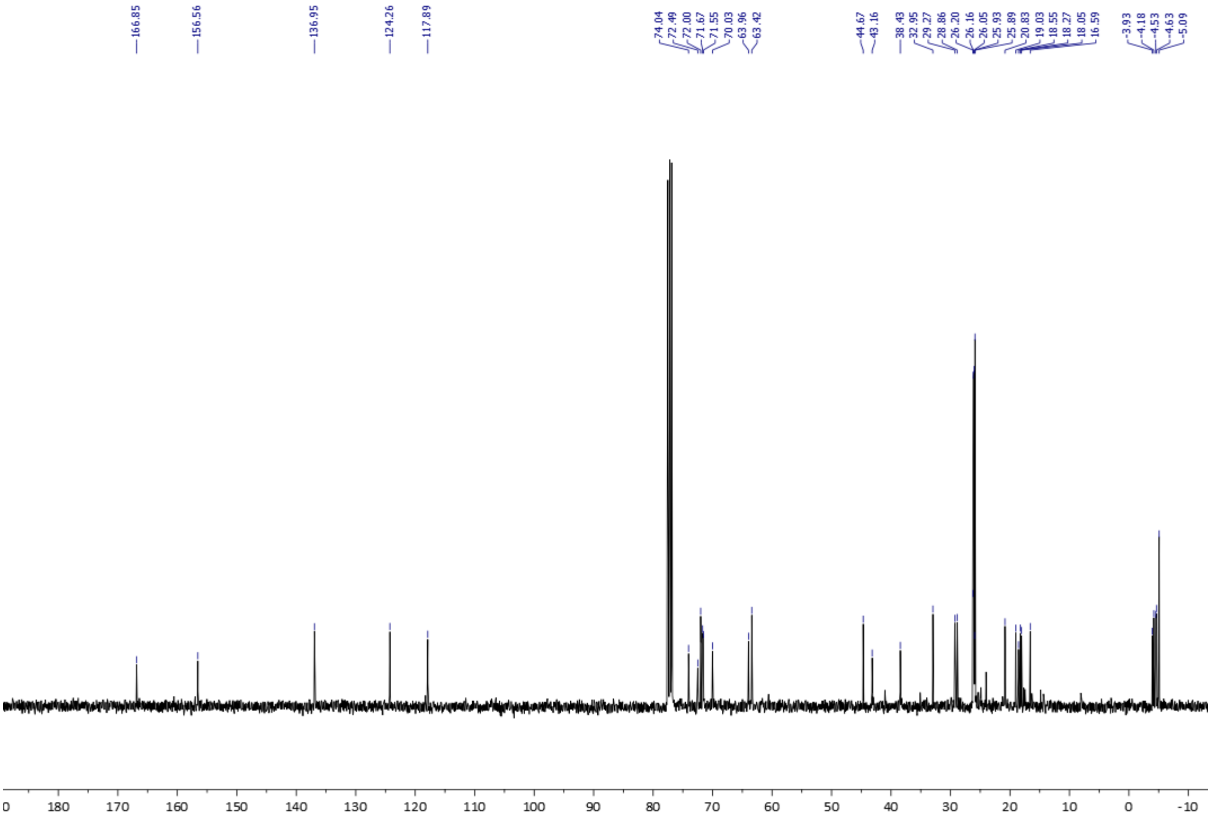

Fig.10: Ester (**25**) ^{13}C-NMR (101 MHz, $CDCl_3$)

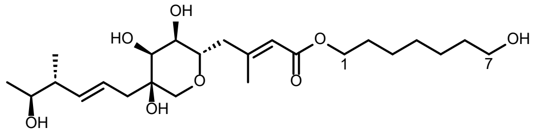

26

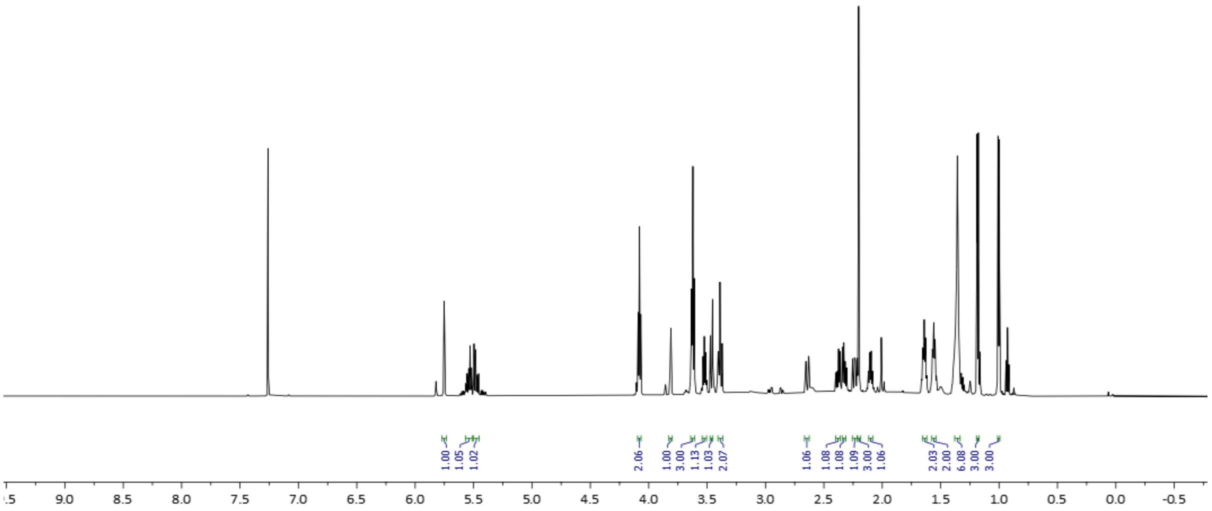

Fig.11: Monate ester (**26**) ^{1}H-NMR (600 MHz, $CDCl_3$)

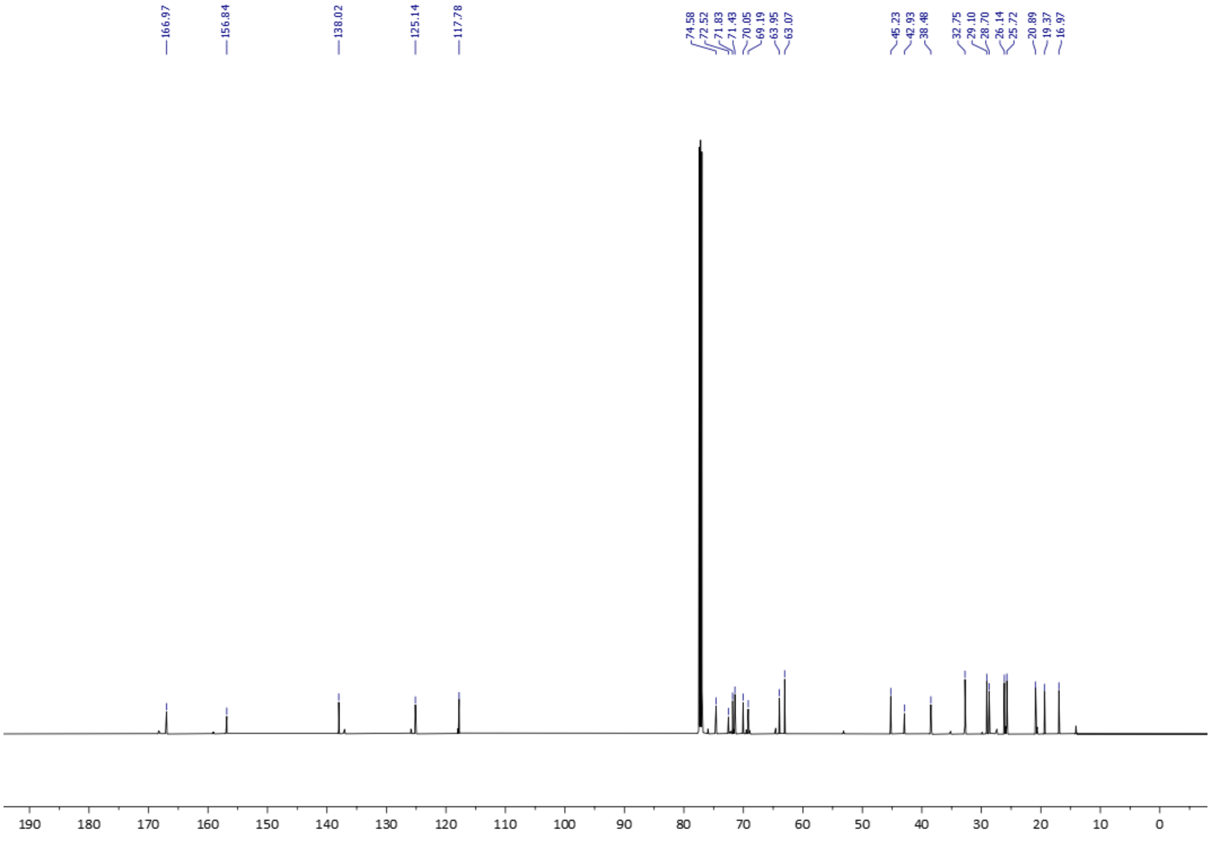

Fig.12: Monate ester (**26**) ^{13}C-NMR (151 MHz, $CDCl_3$)

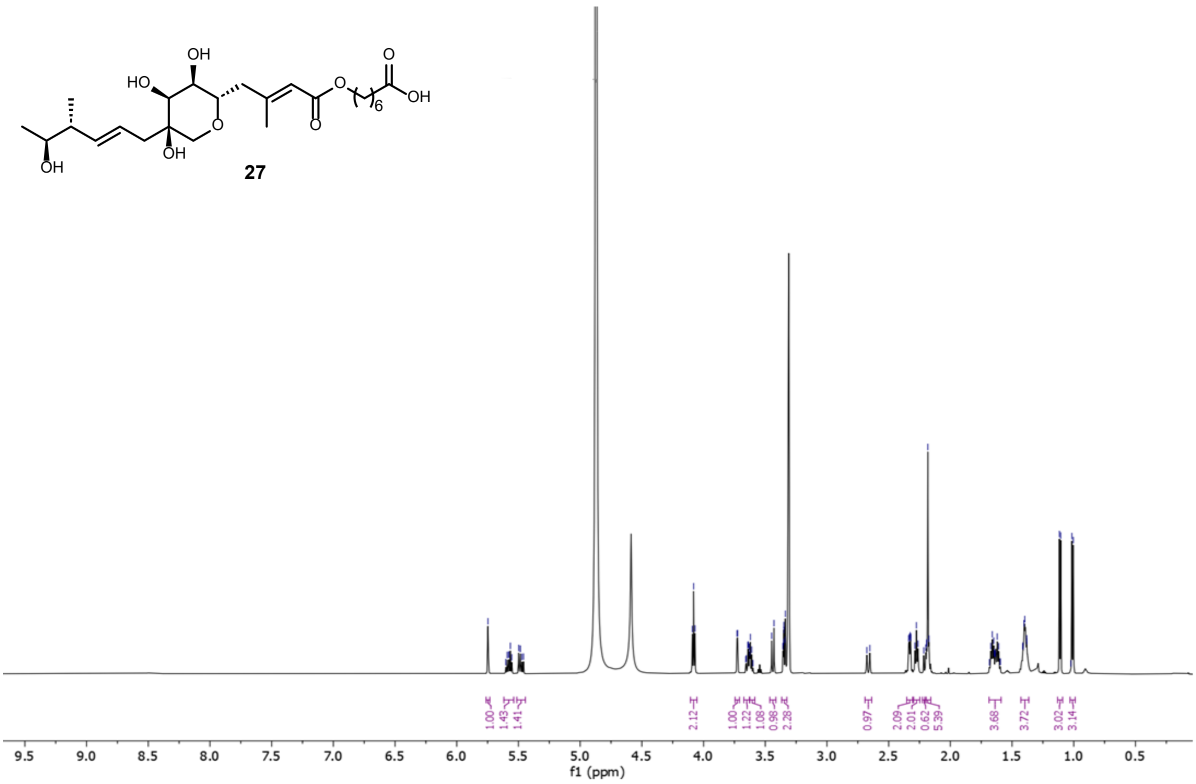

Fig.13: C_7-desepoxy-PA-B (**27**) ^{1}H-NMR (600 MHz, CD_3OD)

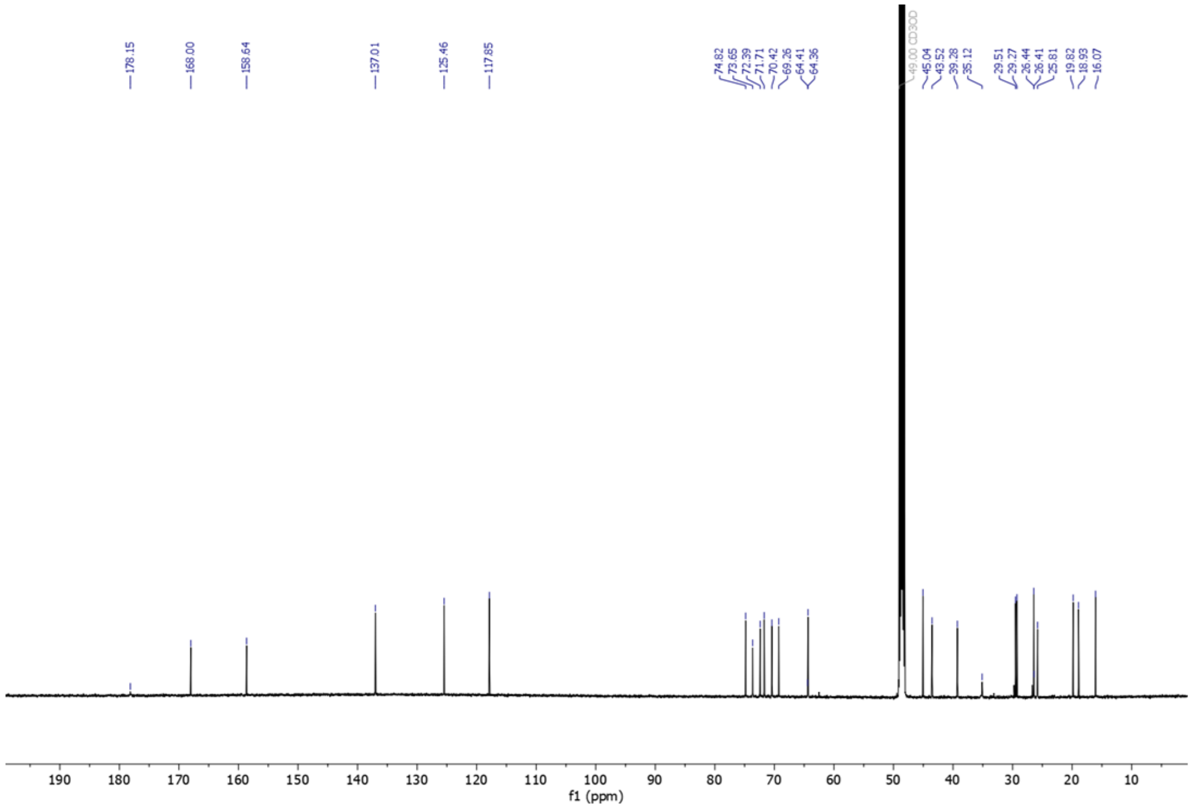

Fig.14: C_7-desepoxy-PA-B (**27**) ^{13}C-NMR (151 MHz, CD_3OD)

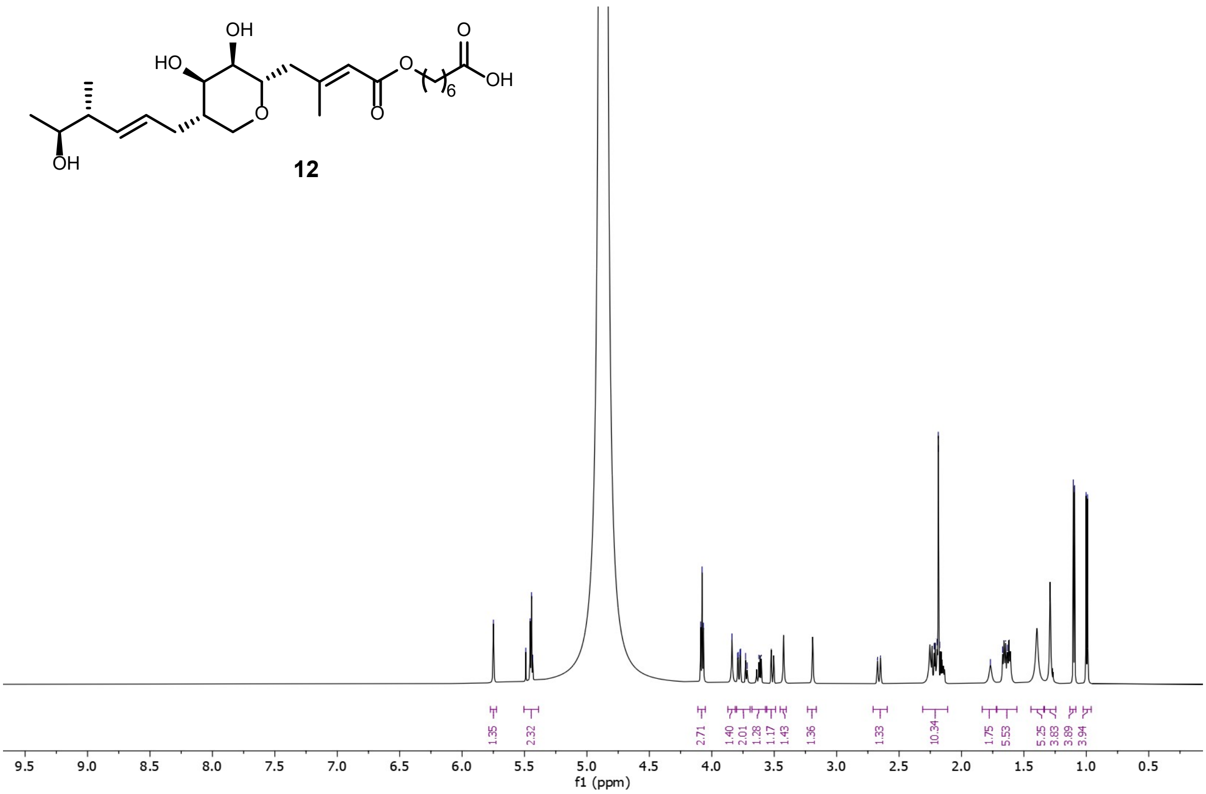

Fig.15: C_7-desepoxy-PA-C (**12**) ^{1}H-NMR (600 MHz, CD_3OD)

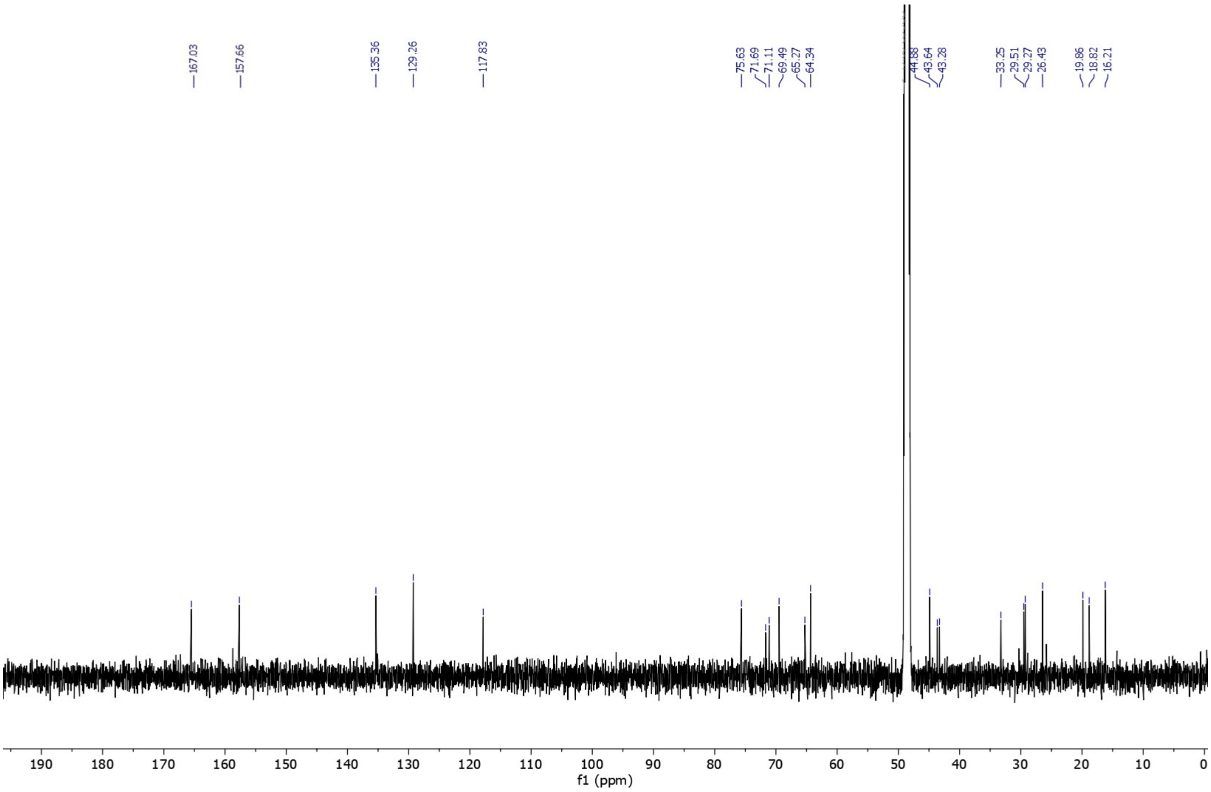

Fig.16: C7-desepoxy-PA-C (12) ^{13}C-NMR (151 MHz, CD_3OD)

5. Reference

1. L. Wang, Z. Song, P. R. Race, J. Spencer, T. J. Simpson, M. P. Crump and C. L. Willis, *Chem. Sci.*, 2020, **11**, 5221-5226.
2. S.-S. Gao, J. Hothersall, J. e. Wu, A. C. Murphy, Z. Song, E. R. Stephens, C. M. Thomas, M. P. Crump, R. J. Cox and T. J. Simpson, *J. Am. Chem. Soc.*, 2014, **136**, 5501-5507.
3. J. P. Clayton, P. J. O'Hanlon and N. H. Rogers, *Tetrahedron Lett.*, 1980, **21**, 881-884.
4. J. P. Clayton, P. J. O'Hanlon, N. H. Rogers and T. J. King, *J. Chem. Soc., Perkin Trans. 1*, 1982, 2827-2833.
5. C. Kuroda, P. Theramongkol, J. R. Engebrecht and J. D. White, *J. Org. Chem.*, 1986, **51**, 956-958.
6. G. F. Kaufmann, R. Sartorio, S. H. Lee, J. M. Mee, L. J. Altobell, 3rd, D. P. Kujawa, E. Jeffries, B. Clapham, M. M. Meijler and K. D. Janda, *J. Am. Chem. Soc.*, 2006, **128**, 2802-2803.
7. F. F. d. P. Hilário, R.C.; Silveira, M.L.T.; Viana, G.H.R.; Alves, R.B.; Pereira, J.R.C.S.; Silva, L.M.; de Freitas, R.P.; de Pilla Varotti, F., *Chem. Biol. Drug Des.*, 2011, **78**, 477-482.
8. N. Amara, R. Mashiach, D. Amar, P. Krief, S. A. Spieser, M. J. Bottomley, A. Aharoni and M. M. Meijler, *J. Am. Chem. Soc.*, 2009, **131**, 10610-10619.
9. L. Wang, A. Parnell, C. Williams, N. A. Bakar, M. R. Challand, M. W. van der Kamp, T. J. Simpson, P. R. Race, M. P. Crump and C. L. Willis, *Nat. Catal.*, 2018, **1**, 968-976.
